# Mapping cell diversity and dynamics in inflammatory temporomandibular joint osteoarthritis with pain at single-cell resolution

**DOI:** 10.1172/jci.insight.184379

**Published:** 2025-02-10

**Authors:** Supawadee Jariyasakulroj, Yang Shu, Ziying Lin, Jingyi Chen, Qing Chang, Pao-Fen Ko, Jian-Fu Chen

**Affiliations:** 1 Center for Craniofacial Molecular Biology, Ostrow School of Dentistry of USC, University of Southern California, Los Angeles, California, USA.; 2 Department of Masticatory Science, Faculty of Dentistry, Mahidol University, Bangkok, Thailand.

**Keywords:** Cell biology, Inflammation, Behavior, Bioinformatics, Osteoarthritis

## Abstract

Temporomandibular joint (TMJ) osteoarthritis with pain is a highly prevalent disorder affecting patients’ quality of life. A comprehensive understanding of cell type diversity and its dynamics in painful TMJ osteoarthritis (TMJOA) is lacking. Here, we utilized an inflammatory TMJOA mouse model via intra-articular injection of CFA. TMJOA mice exhibited cartilage remodeling, bone loss, synovitis, increased osteoarthritis (OA) score, and orofacial pain, recapitulating hallmark symptoms in patients. Single-cell transcriptomic profiling of the TMJ was performed in conjunction with mouse genetic labeling, tissue clearing, light sheet and confocal 3D imaging, multiplex RNAscope, and immunodetection. We visualized, reconstructed, and analyzed the distribution and density of nociceptive innervation of TMJ at single-axon levels. We systematically mapped the heterogeneity and anatomical position of blood endothelial cells, synovial fibroblasts, and immune cells, including *Cx3cr1*-positive barrier macrophages. Importantly, TMJOA mice exhibited enhanced neurovascular coupling, sublining fibroblast hyperplasia, inflammatory immune cell expansion, disrupted signaling-dependent cell-cell interaction, and a breakdown of the sandwich-like organization consisting of synovial barrier macrophages and fibroblasts. By utilizing a mouse model with combined TMJ pain history and OA, we reveal the cellular diversity, anatomical structure, and cell dynamics of the TMJ at single-cell resolution, which facilitate our understanding and potential targeting of TMJOA.

## Abstract 2

We mapped cell diversity and dynamics in inflammatory temporomandibular joint osteoarthritis with pain at single cell resolution.

## Introduction

Temporomandibular disorders (TMDs) are a group of more than 30 human disorders associated with the temporomandibular joint (TMJ), muscles of mastication, and surrounding tissues. TMDs are common, with over 70% of the human population reporting symptoms (1). About 15% of TMDs present as an aggressive disease that is recalcitrant to therapies and leads to chronic pain, making TMDs the second (after chronic low back pain) most common musculoskeletal pain condition. Arthrogenous TMD is a joint-related condition associated with inflammation, dysfunction, and degeneration of the hard or soft tissues within the TMJ. The most common and debilitating forms of arthrogenous TMD are TMJ arthritis and TMJ osteoarthritis (TMJOA), both of which substantially impact the quality of life because of jaw pain and limited jaw movement (2). Localized TMJ arthritis, synovitis, or capsulitis is characterized by joint pain accompanied by clinical signs of inflammation or infection in the absence of substantial bone changes. Localized osteoarthritis (OA), or primary TMJOA, is a degenerative joint disorder that affects only the TMJ and does not involve other systemic joints. Clinical features of TMJOA include the deterioration of articular tissue along with concomitant bony changes in the condyle and articular eminence (3). The characteristics of TMJOA include progressive osseous changes and inflammation in synovial tissue (4, 5). Patients with TMJOA usually have a history of joint pain and joint sounds during jaw movement (6, 7). However, the symptoms can remain subclinical until later stages of degeneration. Although pain is a key reason why patients seek medical treatment, what causes pain in TMDs and how to effectively treat it remain poorly understood (8–10).

The treatment strategies for TMJOA aim at relieving pain, preventing cartilage and subchondral bone destruction, and restoring joint functions. Current clinical practices for TMJOA are invasive joint replacement or conservative therapy, including nonsteroidal antiinflammatory drugs (NSAIDs), splints, and visco-supplementation such as intra-articular injection of hyaluronic acid (11–13). However, 30%–40% of patients are resistant to the first-line NSAID treatment, whose whole-body administration presents side effects with increased risk of gastrointestinal and cardiovascular complications, limiting its general usage (14, 15). Available therapies can alleviate mild to moderate joint pain to a certain degree. Overall, effective disease-modifying OA medications and relief from severe TMJ arthritis pain remain an unmet medical need. The challenges of effectively treating painful TMJOA are, in part, due to a limited understanding of its pathophysiology. TMJOA has multifactorial etiologies, involving different tissues and cell types, such as temporal and mandibular bones, cartilage, osteoclasts, synovium, ligaments, muscles, tendons, and multiple immune cells. Our understanding of TMJOA has been hampered by a limited emphasis on comprehensive and integrated analyses of cellular diversities and functions in the TMJ (4, 5). Few studies have combined findings in multiple cells and their dynamic interactions with their niche environment in TMJOA, which prevents the development of effective, evidence-based treatments.

Animal models play a key role in understanding the pathological process of TMJOA and in evaluating new therapeutic strategies. For the past 4 decades, TMJOA animal models have been extensively used and divided into induced, naturally occurring, and genetically modified models (4, 5, 16). Induced models include the intra-articular injection of inflammatory chemicals (such as complete Freund’s adjuvant [CFA]) (17–20) and surgical trauma such as disc perforation and partial discectomy (21–23). It is technically challenging to perform TMJ surgery in rodent models, especially in the context of pain behaviors with intrinsic variabilities. Therefore, chemically induced inflammatory TMJOA represents a practicable rodent model to recapitulate both TMJ arthritis and OA pathologies and pain behaviors for the assessment of therapeutic strategies (5, 24). It is important to comprehensively characterize TMJ arthritis and pain at morphological, pathological, molecular, cellular, and behavioral levels in these rodent models.

Here, we established a CFA-induced inflammatory TMJOA mouse model with the characterization of synovitis, chondrocyte remodeling, subchondral bone loss, and pain behaviors. We combined single-cell RNA sequencing (scRNA-Seq), 3D imaging, multiplex immunodetection, and highly resolved in situ analysis to catalog the cell types within the TMJ. We defined sensory nerve, blood vessel, macrophage, and fibroblast populations that are enriched in the synovium and retrodiscal tissue (RDT) of TMJs. We revealed cellular dynamics involved in the progression from painful TMJ arthritis to TMJOA, supporting a model of TMJ synovium-driven peripheral sensitization related to pain and bone degeneration. This single-cell atlas provides a comprehensive view of cellular diversities, anatomical position, and dynamics in inflammatory TMJOA associated with pain.

## Results

### Inflammatory TMJOA mouse model.

**Figure 1:**
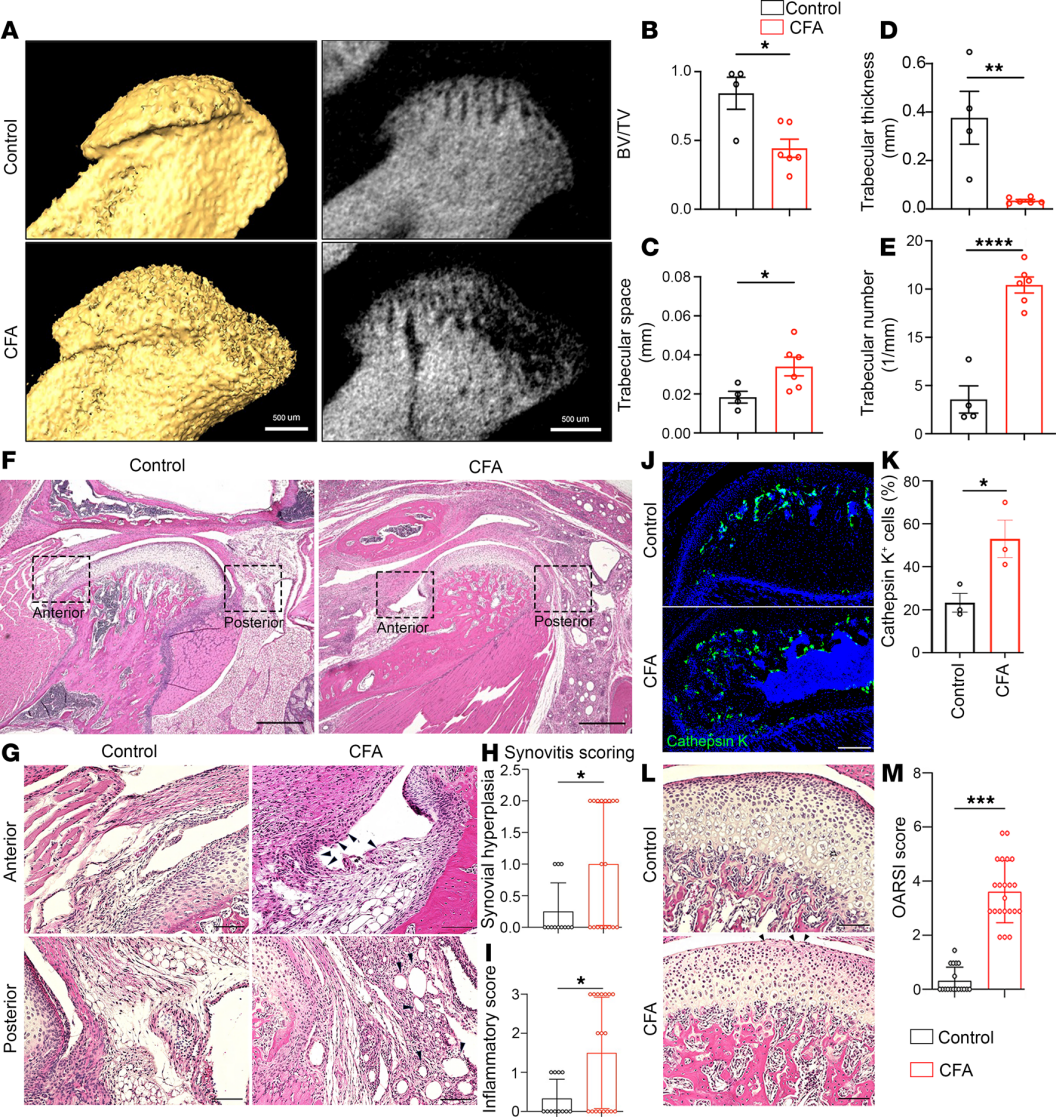
TMJ OA-like defects in CFA intra-articular injection mice. (**A**) Micro-CT of live images of a sagittal view of the mandibular condyle. Scale bar: 500 μm. (**B**–**E**) Quantification analysis of microarchitecture parameters of the subchondral bone. Values represent mean ± SD, *P < 0.05, **P < 0.01, ****P < 0.0001 (*n* = 4 control mice, *n* = 6 CFA-injected mice). BV/TV, bone volume/total volume; 1/mm, 1 trabecular number per mm region. (**F**) H&E staining of sagittal TMJ sections. Black dashed boxes indicate the anterior and posterior synovial tissue around the condyle. Scale bar: 500 μm. (**G**) TMJ synovitis tissue histopathology from boxed regions in **F**. The TMJ of CFA-injected mice presented features of OA-like defects, including hyperplastic epithelial lining (black arrows in anterior area) and immune cellular infiltration caused by inflammation (black arrows in posterior area). Scale bar: 100 μm. (**H** and **I**) Quantification of TMJ synovitis evaluated by Synovitis Scoring System with 2 assessment criteria: synovial hyperplasia (**H**) and inflammatory infiltrate (**I**). Values represent mean ± SD. *P < 0.05 (*n* = 12 sections from 3 control mice, *n* = 18 sections from 4 CFA-injected mice). (**J**) Immunofluorescence staining of Cathepsin K (green) in mandibular condyles. Scale bar: 100 μm. (**K**) Quantification of Cathepsin K^+^ osteoclast cells in subchondral bone of the TMJ condylar head. Values represent mean ± SD calculated by Student’s *t* test. *n* ≥ 3 mice, *P < 0.05. (**L**) H&E staining of sagittal TMJ condylar cartilage part from sections in **F**. Arrowheads indicate uneven surface and loss of fibrous layer in the CFA group. Note the unclear borders between cartilage and subchondral bone, uneven cartilage surfaces, and decreased hypertrophic layer thickness in the CFA group. Scale bar: 100 μm. (**M**) Quantification of Osteoarthritis Research Society International (OARSI) score in TMJs. Data are represented as mean ± SEM calculated by Student’s *t* test. *n* ≥ 3 mice, ***P < 0.001.

Inflammation is a well-accepted pathological trigger for joint pain (5, 25). Therefore, we selected inflammatory over surgical models to investigate TMJ degeneration and pain. To establish an inflammatory TMJOA mouse model, we performed intra-articular injection of inflammatory reagent CFA into both sides of the TMJ to induce inflammation. To examine whether CFA could induce degenerative processes in our mouse model, micro-CT imaging was used to analyze bone architecture at 3–4 weeks after CFA intra-articular injection. There was a substantial morphological change in the subchondral bone in CFA groups compared with PBS controls (Figure 1A). CFA-treated TMJ exhibited a substantial reduction in bone volume (bone volume over total volume, BV/TV) and in trabecular thickness (Figure 1, B and D), as well as a robust increase in trabecular space and trabecular number (Figure 1, C and E). Therefore, CFA-injected TMJ displayed bone loss, possibly due to impaired bone-generating osteoblasts or increased bone-resorbing osteoclasts. To investigate cellular mechanisms underlying the bone loss, we used Cathepsin K to label osteoclasts. Our immunohistochemical (IHC) staining showed an increase of Cathepsin K^+^ cells in TMJ subchondral bones in the CFA group compared with controls (Figure 1, J and K). Together, these results showed robust subchondral bone loss because of greater osteoclasts in CFA-injected TMJ.

To further examine the pathologies, we performed H&E staining of the TMJ at 3–4 weeks after CFA intra-articular injection. We focused on the condyle and observed an intact and continuous surface of cartilage; matrix was distributed evenly without deformation in control groups (Figure 1, F and L). However, the top fibrous layer over the articular surface was diminished in CFA-treated TMJ. The continuous surface of subchondral bone in a normal TMJ was also impaired with deformation in CFA-treated TMJ (Figure 1, F and L). We closely examined the anterior and posterior regions of the TMJ and observed inflammation in the soft tissue surrounding TMJ, including synovial hyperplasia and inflammatory cell infiltration in synovial tissue (Figure 1, G–I). We applied Osteoarthritis Research Society International (OARSI) scoring systems and observed a notable increase in OARSI score in CFA-injected TMJ compared with controls (Figure 1, L and M). Together, these results suggest that CFA-induced inflammation resulted in subchondral bone loss, cartilage remodeling, and synovitis in the TMJ, which are consistent with pathological findings in TMJOA.

### Pain behavior and its neural substrate in the TMJOA mouse model.

**Figure 2:**
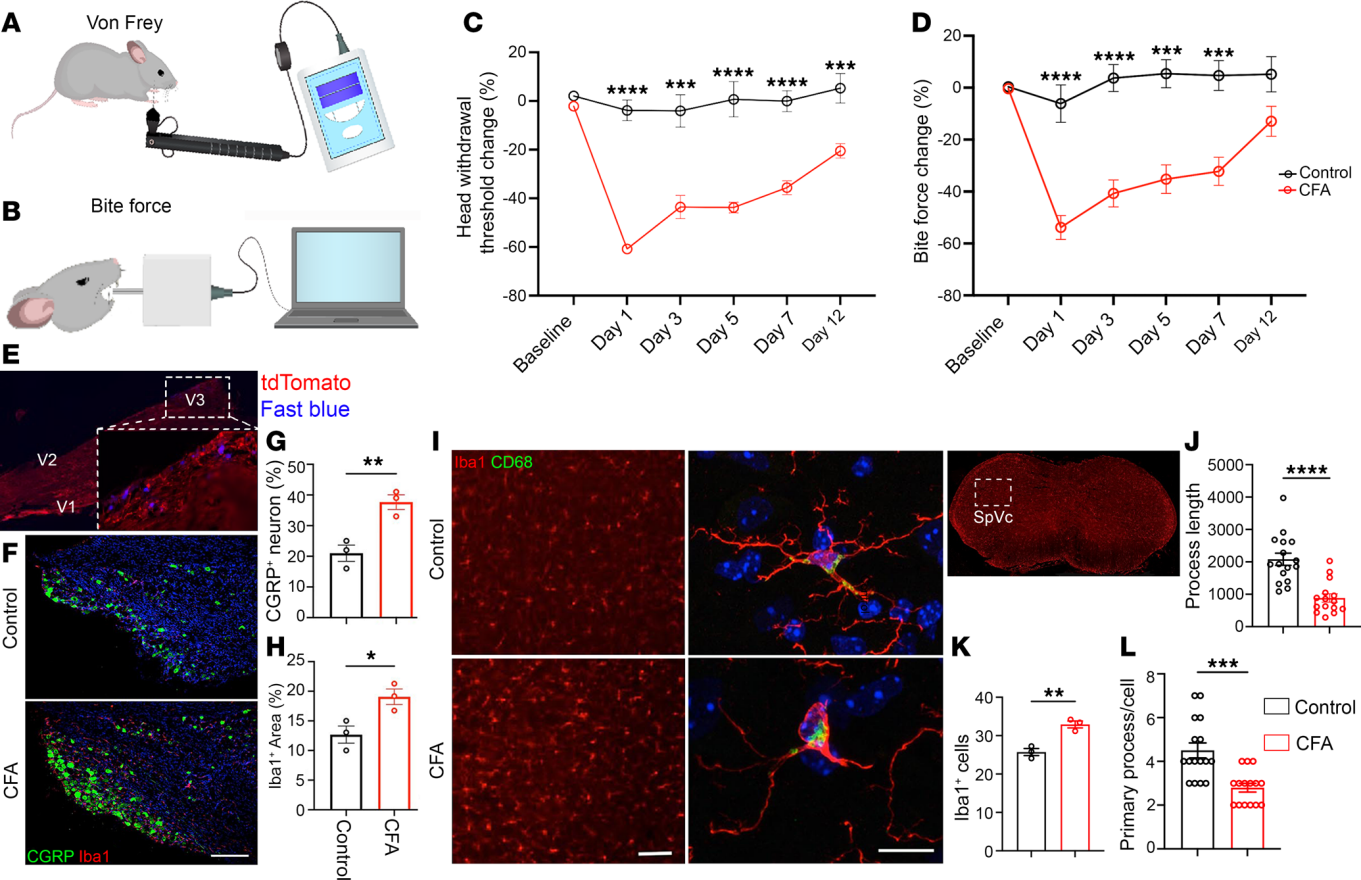
Painful behaviors and neuroimmune response in CFA intra-articular injection mice. (**A**) Diagram of von Frey filament test. (**B**) Diagram of bite force measurement. (**C**) Quantification of head withdrawal threshold measurement at different time points after CFA injection. *N* = 7 mice (control), *n* = 10 mice (CFA). (**D**) Quantification of relative bite force values at different time points. *N* = 7 mice (control), *n* = 10 mice (CFA). (**E**) Confocal imaging of tdTomato TG section after retrograde tracer Fast Blue in the TMJ. V1, V2, V3 represent ophthalmic nerve (V1), maxillary nerve (V2), and mandibular nerve (V3). Original magnification, 4× for original image; 20× for zoomed-in image. (**F**) Immunofluorescence staining of CGRP (green) and Iba1 (red) in V3 section of TG. Scale bar 100 μm. (**G** and **H**) Quantification of the percentage of CGRP^+^ neurons and the percentage of Iba1^+^ area fraction in the V3 of TG. *N* = 3 mice. (**I**) Immunofluorescence staining of spinal trigeminal nucleus caudalis (SpVC) using antibodies against microglia marker Iba1 (red) and microglial activation marker CD68 (green). Scale bars: 100 μm. (**J**) Quantification of the total length of processes per microglia. *N* ≥ 3 mice. (**K**) Quantification of Iba1^+^ cells in SpVC area. *N* = 3. (**L**) Quantification of the number of primary processes per microglia. *N* ≥ 3 mice. All data are represented as mean ± SEM calculated by Student’s *t* test. *n* ≥ 3 mice, *P < 0.05, **P < 0.01, ***P < 0.001, ****P < 0.0001. TG, trigeminal ganglion; CGRP, calcitonin gene-related peptide; Iba1, ionized calcium-binding adapter molecule 1.

The CFA-induced inflammatory TMJ arthritis and osteoarthritis models have been used to study TMJ pain mechanisms (5, 25). To investigate whether the CFA intra-articular injection can induce pain symptoms, we measured nociceptive behaviors using bite force and von Frey filament assays. Our von Frey filament test measures the lowest threshold force that could lead to head withdrawal behavior, indicating pain response and hyperalgesia in the orofacial region (Figure 2A). Reduced head withdrawal threshold could be interpreted as joint tenderness and reduced pressure pain threshold presenting in patients with TMJ pain in TMJ arthritis and OA (26, 27). Longitudinal quantification showed that CFA injection significantly lowered the head withdrawal threshold after the filament probing of TMJ regions (Figure 2C). The TMJ is one of the key structures involved in all jaw movement, such as mouth opening/closing, biting, and chewing. The dominant clinical feature of TMD is chewing or mastication-related pain. Consistent with clinical studies, patients with joint pain had lower bite force compared with healthy individuals (27). In addition, reduced bite force in patients with TMJ pain is correlated with their lower pressure pain threshold (27, 28). Therefore, we used bite force measurement to monitor pain behavior and TMJ function (Figure 2B). Longitudinal quantification showed that CFA injection significantly reduced the bite force (Figure 2D), suggesting TMJ pain and impaired joint function. Together with recent studies (29), bite force and von Frey filament assays provide reliable methods for pain behavior assessment; these orthogonal behavior studies verified the pain behaviors in CFA-induced TMJOA mouse models.

To investigate cellular substrates underlying TMJ pain behaviors, we examined nociceptive innervation of TMJs. To this end, we performed retrograde tracing of TMJ-projecting TG neurons. The tdTomato-positive mice were unilaterally injected with retrograde tracer Fast Blue into the TMJ, followed by TG dissection and histological analysis. We detected Fast Blue signals in sensory neuron cell bodies of specific V3 regions of the TG (Figure 2E), which is consistent with the notion that the lower jaw is innervated by V3 neurons in the TG (5). Nociceptive signals are detected in the TMJ and sent to neuron cell bodies (first-order neurons) in the TG, which are immunoreactive for the CGRP (30). CGRP-containing nerve fibers are found in the joint capsules, disc, and synovial membrane of TMJ, with increased levels of CGRP in synovial fluid of human arthritic TMJs correlating with pain (31, 32). Functional studies in animal models suggest that CGRP is necessary and sufficient for TMD pain (33). CGRP is also involved in the peripheral injury and inflammation, resulting in peripheral sensitization and central pain processing (34). IHC staining of the TG showed that there was an increased CGRP^+^ and Iba1^+^ immune reaction in the TG V3 region (Figure 2, F–H), implying an increase in genes or numbers of nociceptive neurons and macrophages in the peripheral nervous system after CFA injection. The first-order TG neurons synapse with second-order neurons in the spinal trigeminal nucleus caudalis (Sp5C) of the brainstem for nociceptive signal transmission to the brain. We used Iba1 to assess microglia morphology and CD68 to monitor microglial activation. IHC staining of Sp5C showed CFA treatment led to increased expression of CD68 and increased numbers of Iba1^+^ microglia (Figure 2, I and K); primary process numbers and length of individual process per microglia were decreased in the CFA group compared with controls (Figure 2, J and L), indicating microglial activation in the Sp5C of CFA-treated mice. Therefore, CFA treatment causes inflammatory sensitization in TMJs and leads to macrophage and microglial activation in the TG and Sp5C, respectively, providing a neuroimmune substrate for orofacial pain mice.

### Anatomic mapping of TMJ nociceptive innervation.

**Figure 3:**
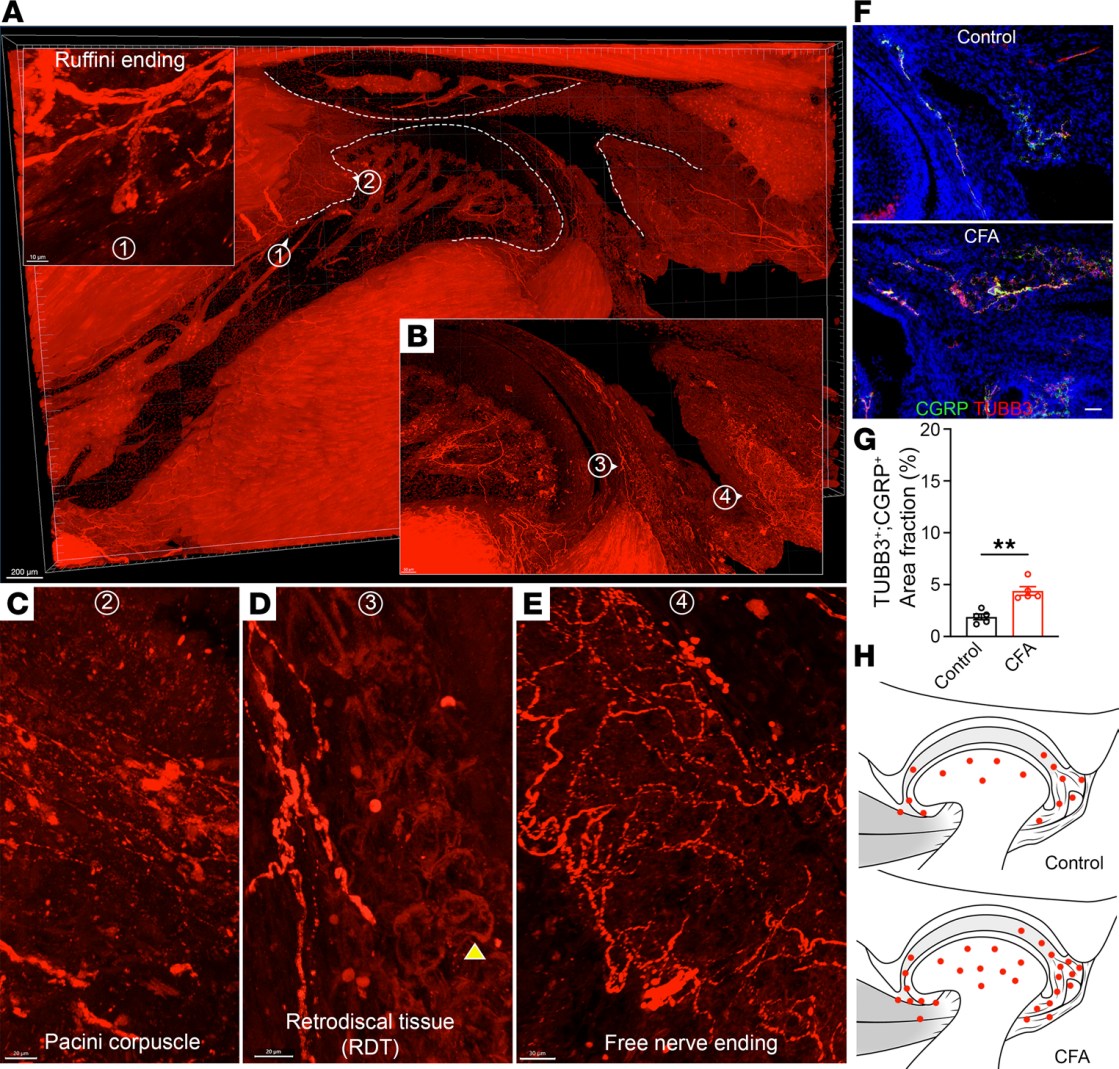
Anatomical distribution of TMJ innervation and increased sensory innervation in CFA mice. (**A**) Confocal imaging of the sagittal section (*Z*-stack of 100 μm) of TMJ from *Na_v_1.8-Cre Ai14* mice. Scale bar: 200 μm. (A-1) Enlarged image represents the Ruffini-like encapsulated ending in the anterior synovial tissue region. Scale bar: 10 μm. (**B**) Enlarged imaging of the posterior region of TMJ section containing extensive innervation in subchondral bone, posterior part of the articular disc, retrodiscal tissue, and synovial tissues. (A-2 and **C**) Pacinian corpuscle-like structure found in the anterior part of the TMJ capsule. Scale bar: 20 μm. (**D**) Innervation of TMJ RDT enriched with fat pad (yellow arrowhead). Scale bar: 20 μm. (**E**) Nerve plexus containing free nerve endings is found in the posterior part of the TMJ capsule. Scale bar: 30 μm. (**F**) Immunofluorescence staining of the superior part of sagittal TMJ sections with antibodies against CGRP (green) and TUBB3 (red). DAPI stains nuclei (blue). Scale bar: 100 μm. (**G**) Quantification of CGRP^+^TUBB3^+^ area fraction surrounding TMJ. *N* = 5. (**H**) Schematic of nerve innervation surrounding TMJ showed an increased innervation in CFA mice, compared with control. All data are represented as mean ± SEM. Student’s *t* test. *n* = 5 mice, **P < 0.01. TUBB3, tubulin beta 3.

To investigate TMJ innervation, we used thymus cell antigen 1–EGFP (Thy1-EGFP) mice (35) subjected to the clear, unobstructed brain/body imaging cocktails and computational analysis tissue clearing of TMJ, followed by confocal time-lapse imaging analysis. Extensive nerve bundles, fibers, and axonal branches were found in the bone, muscle, synovial tissue, and capsule of the TMJ (Supplemental Figure 1A and Supplemental Video 1; supplemental material available online with this article; https://doi.org/10.1172/jci.insight.184379DS1). To specifically label nociceptors, we used *Na_v_1.8*-Cre transgenic mice, in which *Na_v_1.8* encodes a voltage-gated sodium channel expressed only in a subset of sensory neurons, of which more than 85% are nociceptors (36). We generated *Na_v_1.8-Cre Ai14* mice, in which tdTomato fluorescently labels nociceptor sensory neurons. Following this, we performed immunolabeling-enabled 3D imaging of solvent-cleared organs (37) of adult mouse TMJs. Confocal time-lapse imaging showed an extensive innervation of the TMJ after 3D reconstruction (Supplemental Figure 1B and Supplemental Video 2). To examine nociceptors at single axonal levels, we focused on individual tissue sections at the central region of the *Na_v_1.8-Cre;Ai14* mouse TMJ. There was extensive nociceptive innervation in the anterior and posterior synovial regions, RDT, and muscle, with relatively less nociceptor detection in bone marrow (Figure 3A and Supplemental Video 3). Sensory receptors can be classified into 2 major groups, including free nerve endings and encapsulated nerves (38). The free nerve ending plexus is found in the synovial regions and RDT, where they likely serve as the nociceptors for pain sensitization in TMJs (Figure 3, A, B, and E). Within the RDT, nociceptors were found adjacent to the articular fat pad (yellow arrowhead, Figure 3D). In the temporomandibular articular disk, the nociceptor density was higher at the periphery and progressively decreased toward the center (Figure 3B). In addition to free nerve endings, we found 2 encapsulated nerve endings, including Ruffini-like receptors and Pacini-like corpuscles (Figure 3, A and C). The Ruffini ending represents small, spindle-shaped, slowly adapting receptors in connective tissues, whereas the Pacinian corpuscle is an onion ball–like structure. Both encapsulated nerve endings contain mechanoreceptors and play crucial functions in joint mechanic pressure (38). Together, these studies reveal the morphology, anatomic distribution, and density of innervating nociceptors of the TMJ (Figure 3H).

To investigate how joint innervation changes in CFA-induced inflammatory TMJOA, we compared control and CFA-induced TMJOA mice. IHC staining of mouse joint sections was carried out using antibodies against pan-neuron marker TUBB3 and CGRP, a marker of sensory afferent activation in the respective tissue during a pain condition (30). In the superior regions including RDT, there was an increase of TMJ innervation in CFA mice compared with control (Figure 3F), as reflected by the significant upregulation of the TUBB3^+^CGRP^+^ double-positive area in CFA mice compared with controls (Figure 3G). In addition, CFA mice exhibited an induced innervation in the anterior and posterior synovial tissue adjacent to the condyle of the TMJ in comparison with controls (Supplemental Figure 1, C–F). We did not notice the robust nerve induction in the temporomandibular bone marrow and the central regions of the articular disc, suggesting that RDT and synovial tissue might be the main articular tissues where pain can be elicited. Together, these results suggest that there is a robust induction of sensory innervation in TMJOA mice, which could be responsible for joint peripheral sensitization leading to pain behavior.

### The scRNA-Seq analysis of major cell types in normal and OA TMJ.

**Figure 4:**
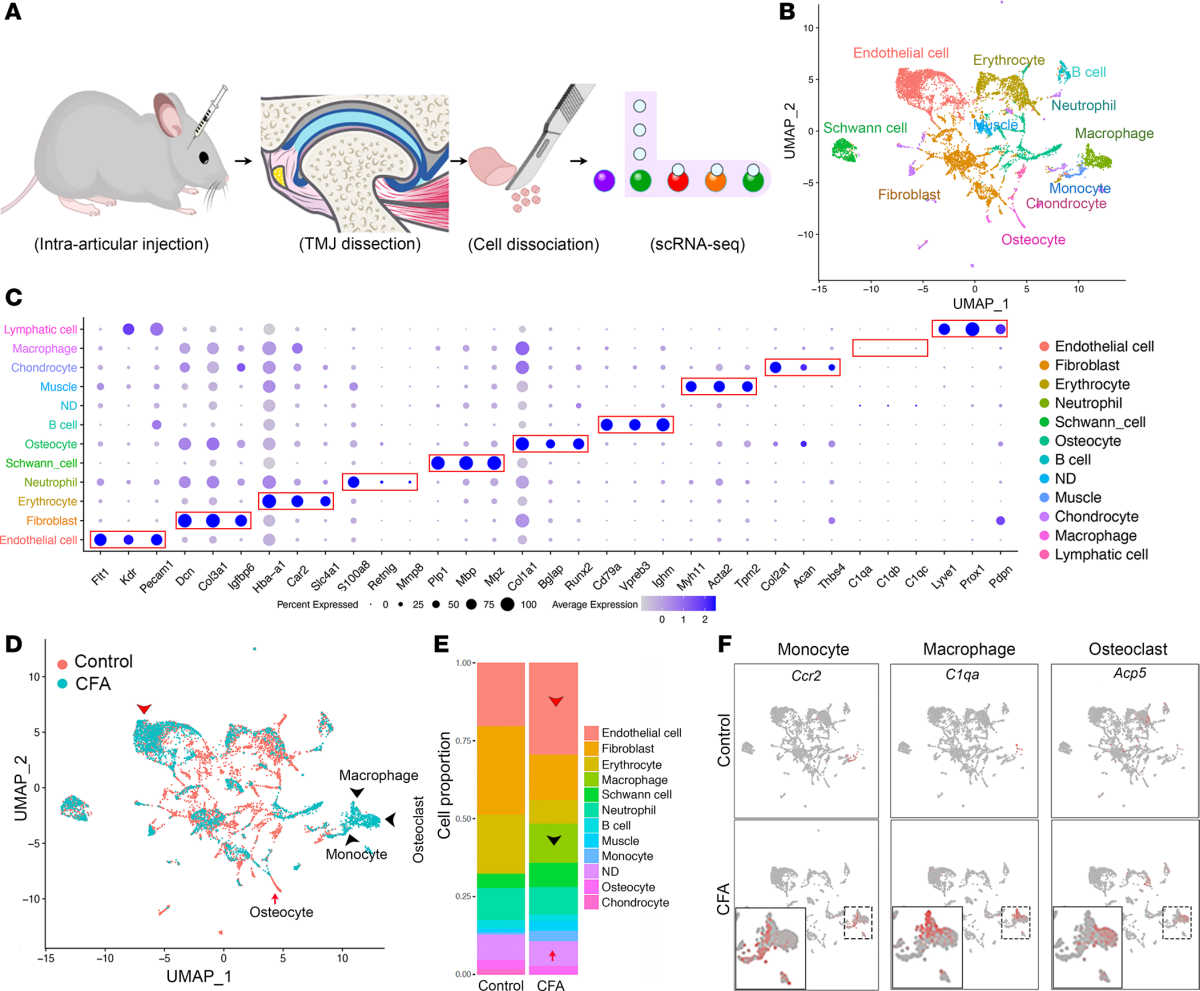
scRNA-Seq analysis of all cells in the TMJ of control and CFA mice. (**A**) Schematic diagram of TMJ inoculation and experimental procedure of single-cell analysis. (**B**) Uniform manifold approximation and projection (UMAP) visualization of major cell types highlighted with different colors in TMJ. (**C**) Dot plot depicting selected markers enriched for each cell population within the TMJ. (**D**) UMAP plot for both control (red) and CFA (blue) with the major cell types. Red arrow indicates the reduced osteocyte population in CFA group, while black arrowheads represent the increased monocyte, macrophage, and osteoclast subsets in CFA group compared with control. The red arrowhead indicates an increased endothelial cell population in the CFA group compared with the control. (**E**) Proportion of each cell cluster in CFA group versus control. Red arrow indicates the reduced osteocyte population, and black arrowheads represent the increased macrophages in CFA group compared with control. The red arrowhead indicates an increased endothelial cell population in the CFA group compared with the control. (**F**) Feature plots showing the expression of *Ccr2* (monocytes), *C1qa* (macrophages), and *Acp5* (osteoclasts), which are distributed closely but are separated from each other in UMAP plot and are increased in CFA group compared with control. Normalized expression levels for each cell are color-coded and overlaid onto the UMAP plot. Myh11, myosin heavy chain 11; Acta2, α–smooth muscle actin; ND, not determined.

To investigate cellular mechanisms underlying the joint degeneration in TMJOA mice, we systematically examined major cell types in the TMJ using the scRNA-Seq approach. The TMJ samples were taken at 3 weeks post–intra-articular inoculation of PBS or CFA from 2-month-old female mice (Figure 4A). The time point corresponds to the stage with confirmed OA pathologies. Female mice were chosen because females are disproportionally affected by TMJ dysfunction, reporting jaw pain at twice the rate of male humans (39). FACS analysis of single-cell suspension revealed around 47.9% immune cells among 79% live cells from CFA-treated joints (Supplemental Figure 2, A and B). To avoid the immune cell dominance, we performed CD45^+^ immune cell reduction after single-cell preparation. To that end, PBS and CFA joint samples contained ~10.9% and ~20.8% immune cells, respectively (Supplemental Figure 2C). Using Seurat 3 R package, we obtained 7,667 cells (median of 2,345 genes per cell) in control and 7,856 cells (median of 3,421 genes per cell) in CFA joints. After unbiased clustering of gene profiles, cell type identity was defined based on the top differentially expressed genes and expression of known cell type marker genes (Figure 4C). We identified 11 primary cell types (Figure 4, B and C), including endothelial cell (*Flt1*, *Kdr*, *Pecam1*), fibroblast (*Dcn*, *Col3a1*, *Igfbp6*), erythrocyte (*Hba-a1*, *Car2*, *Slc4a1*), neutrophil (*S100a8*, *Retnlg*, *Mmp8*), Schwann cell (*Plp1*, *Mbp*, *Mpz*), osteocyte (*Col1a1*, *Bglap*, *Runx2*), B cell (*Cd79a*, *Vpreb3*, *Ighm*), muscle (*Myh11*, *Acta2*, *Tpm2*), chondrocyte (*Col2a1*, *Acan*, *Thbs4*), macrophage (*C1qa*, *C1qb*, *C1qc*), and lymphatic cell (*Lyve1*, *Prox1*, *Pdpn*). These cell clusters and their constituent cell types were relatively well separated (Figure 4B), suggesting that our scRNA-Seq data are of high integrity.

By combining control and CFA samples using Seurat, we observed an increase in the percentage of endothelial cells in the CFA group. Compared with PBS control, the CFA group tended to have a decreased trend in chondrocytes (*Col2a1*, *Acan*) and in osteocytes (*Bglap*, *Dmp1*) in feature plot (Supplemental Figure 2D) as well as bar graph and UMAP analyses (Figure 4, D and E, red arrow), which is consistent with chondrocyte remodeling and bone loss phenotypes in CFA-treated TMJs. In contrast, CFA group had a drastic increase in immune cells (Figure 4, D and E), which is consistent with the FACS analysis (Supplemental Figure 2C). The monocytes (*Ccr2*), macrophages (*C1qa*), and osteoclasts (*Acp5*) were in a relatively close position but had clear separation in UMAP analysis and feature plots (Figure 4, D and F). Importantly, these 3 types of immune cells were robustly increased in CFA group compared with controls, which is consistent with inflammation and synovitis phenotypes in TMJOA mice. Our scRNA-Seq identified major cell types in the TMJ and revealed their dynamic changes in TMJ arthritis with pain.

### Vascular endothelial cell heterogeneity and increased neurovascular coupling in TMJOA.

**Figure 5:**
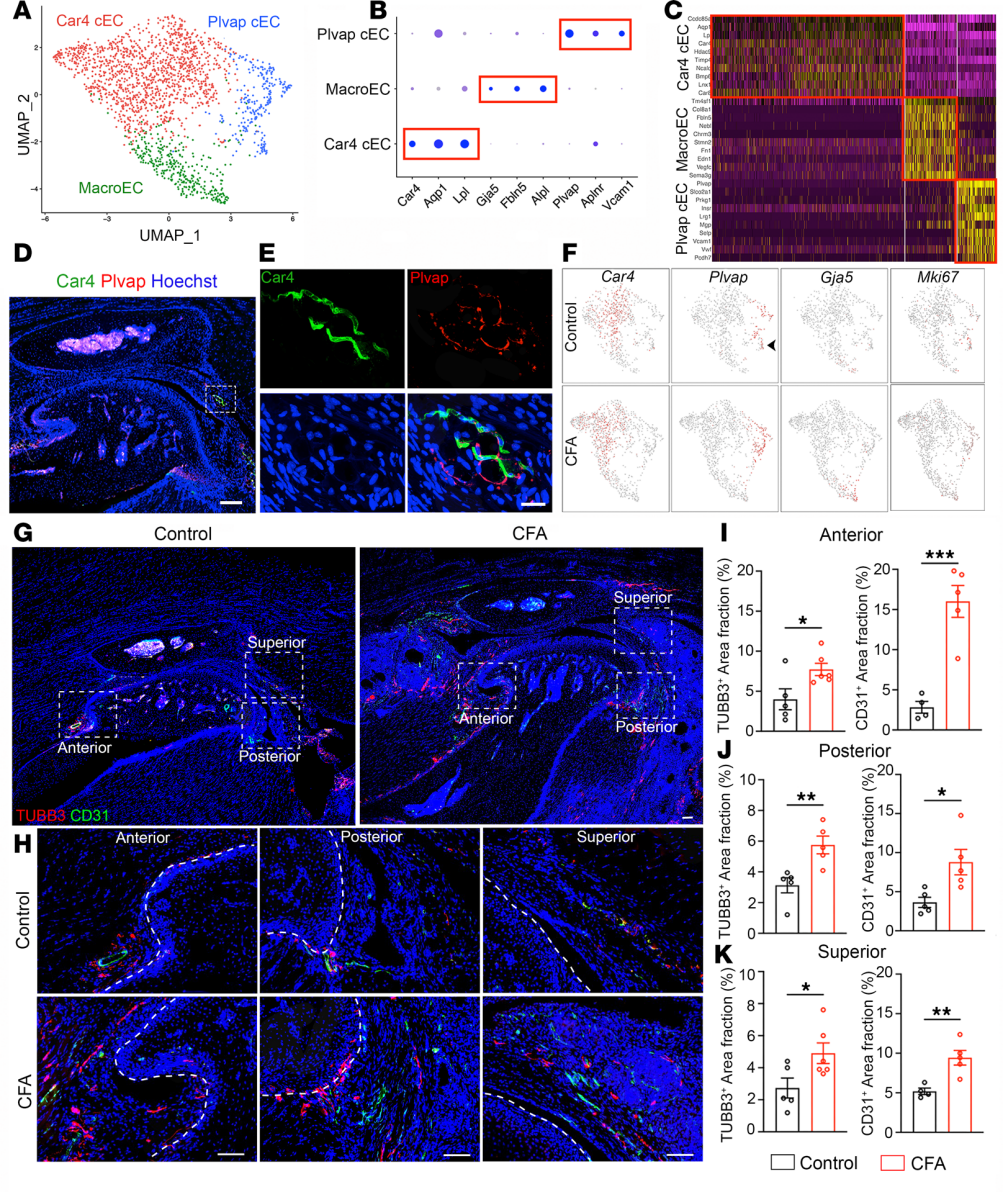
Endothelial cell heterogeneity and neurovascular induction in control and CFA TMJ. (**A**) UMAP plot of endothelial cell clusters in adult mouse TMJ. (**B** and **C**) Dot plot and heatmap of signature genes in different endothelial cell clusters, including Plasmalemma vesicle-associated protein capillary endothelial cell (Plvap cEC), macrovascular endothelial cell (MacroEC), and carbonic anhydrase 4 capillary endothelial cell (Car4 cEC). (**D** and **E**) Confocal imaging of TMJ sagittal sections stained with antibodies against Car4 (green) and Plvap (red). DAPI stains nuclei (blue). Images in **E** are enlargements of boxed TMJ RDT in **D**. Scale bar: 100 μm in **D** and 10 μm in **E**. (**F**) Feature plots showing the expression of indicated genes that are overlaid on the UMAP plot. Black arrowheads indicate the Plvap capillary endothelial cells expressing proliferation marker Mki67. (**G** and **H**) Confocal imaging of sagittal TMJ sections stained with antibodies against TUBB3 (red) and CD31 (green). DAPI stains nuclei (blue). Images in **H** (20× objective) are enlargements of boxed regions of TMJ in **G** (4× objective) at the anterior, posterior, and superior regions. Scale bars: 100 μm. (**I**–**K**) Quantification of the area fraction of TUBB3^+^ neural and CD31^+^ vasculature area fraction in the TMJ area. *N* ≥ 4 mice. All data are represented as mean ± SEM. Student’s *t* test. *n* ≥ 4 mice, *P < 0.05, **P < 0.01, ***P < 0.001.

Angiogenesis is increased during the development of knee OA and leads to ossification in osteophytes and in deep layers of articular cartilage (40). Angiogenesis potentially contributes to structural damage and pain in knee OA (40). Although developmental origin, structural, and functional differences between knee and temporomandibular joints have been noted (41, 42), the vasculature heterogeneity in the TMJ remains poorly understood. To investigate vascular endothelial tissues in our joint dissections, we reclustered the endothelial populations and obtained 3 major cell types present in TMJs (Figure 5A). Analysis of enriched genes for each cluster allowed us to assign potential identities to each type, including 1 macrovascular endothelial cell (MacroEC marked by *Gja5*, *Fbln5*, *Alpl*) and 2 capillary endothelial cells (cECs). We named Car4 cEC since Carbonic anhydrase 4 (*Car4*) is its most specific marker, along with *Aqp1* and *Lpl*. The remaining cECs express Plasmalemma vesicle-associated protein (*Plvap*) and are named Plvap cECs (*Plvap*, *Aplnr*, *Vcam1*) (Figure 5, B and C). Plvap-expressing ECs include Mki67-expressing proliferative ECs (Figure 5F) and support the possibility that Car4 cEC originates from Plvap cEC. Immunostaining revealed the enriched localization of Car4 cECs and Plvap cECs in the anterior and posterior synovial tissues, as well as in the RDT regions of the TMJ (Figure 5D). Interestingly, these 2 capillary ECs are mosaics with intermingled distribution in RDT (Figure 5E). Together, these data provide an overview of the cellular heterogeneity, distribution, and organization of endothelial cells in the TMJ.

To investigate the change of angiogenesis during TMJ arthritis, we used CD31 (also known as PECAM-1) pan–endothelial cell marker to label all endothelial cells. Blood vessels and sensory nerves are often coupled, and sensory nerves are reported to grow along new blood vessels in knee OA joints (40). Therefore, we used TUBB3 to label neurons in conjunction with CD31-positive blood vessels. We performed IHC staining of sagittal TMJ sections and found neurovascular coupling enriched in the anterior, posterior, and superior regions, as well as in the subchondral bone marrow area of the TMJ (Figure 5G). The neurovascular regions were rarely found in the central regions of articular disk or cartilage but were progressively enriched in the peripheral regions with intermingled distribution in the RDT and capsule regions (Figure 5G). There were no notable changes of neurovascular structure in the subchondral bones between control and CFA groups. In contrast, CFA mice exhibited a significant increase in the TUBB3- and CD31-immune reacting area compared with controls in the examined regions, including anterior, posterior, and superior parts of the TMJ (Figure 5, G–K). Together, these results suggest an increase in blood vessels coupled with sensory innervation in inflammatory joint tissue during TMJOA.

### Synovial fibroblast heterogeneity and anatomic structure in normal and OA TMJ.

**Figure 6:**
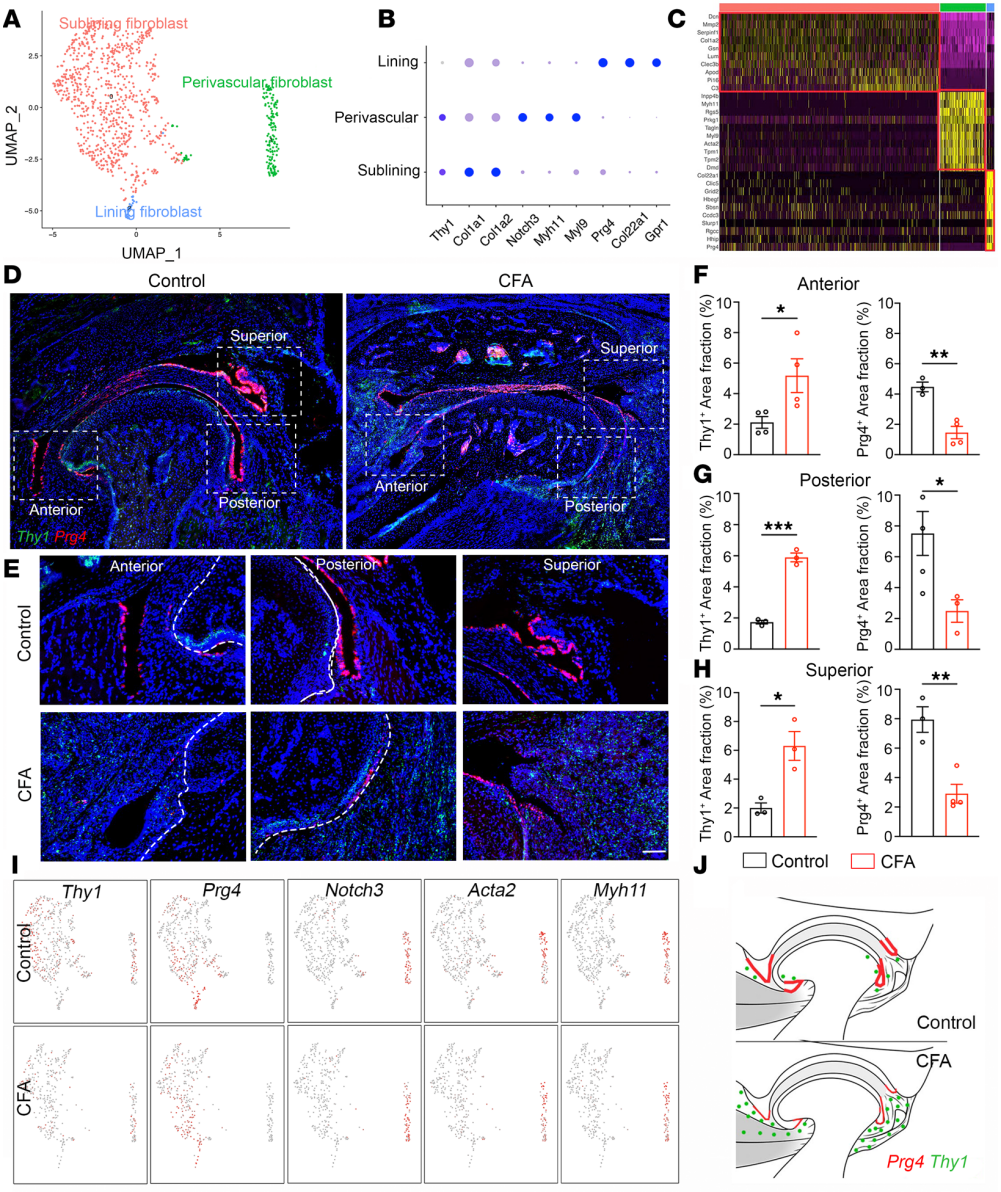
Synovial fibroblast heterogeneity and anatomical locations in control and CFA TMJ. (**A**) UMAP plot of fibroblast cell clusters in adult mouse TMJ. (**B** and **C**) Dot plot and heatmap of signature genes in different fibroblast cell clusters including lining, perivascular, and sublining fibroblasts. (**D** and **E**) Confocal imaging of TMJ sagittal sections after RNAscope staining of *Thy1* (green) and *Prg4* (red). DAPI stains nuclei (blue). Images in **E** (20× objective) are enlargements of boxed TMJ regions in **D** (4× objective). Scale bars: 100 μm. (**F**–**H**) Quantification of the area fraction of *Thy1*^+^ sublining fibroblast and *Prg4*^+^ lining fibroblast area fraction in the TMJ area. *N* ≥ 3 mice. (**I**) Feature plots showing the expression of indicated genes that are overlaid on the UMAP plot. (**J**) Schematic overview of expanded *Thy1^+^* sublining fibroblasts and reduced *Prg4^+^* expression in synovial lining fibroblasts in CFA-induced TMJOA with pain. All data are represented as mean ± SEM. Student’s *t* test, *N* ≥ 3 mice. *P < 0.05, **P < 0.01, ***P < 0.001. *Prg4*, proteoglycan 4.

The synovial membrane is an important structure covering the inner surface of the joint capsule. It functions in the production, secretion, and resorption of synovial fluids. In patients, experiencing TMJ pain is often associated with inflammation in the synovial membrane and underneath the synovium, leading to synovitis (43). In rheumatoid arthritis (RA), the synovial tissue in knee joints undergoes profound hyperplasia, becomes inflamed and invasive, and damages adjacent cartilage and bone (44). To determine fibroblast heterogeneity in TMJs, we reclustered the fibroblast populations and obtained 3 major cell types present in the TMJ (Figure 6A). Analysis of enriched genes for each cluster allowed us to assign potential identities to each type, including 1 lining fibroblast (*Prg4*, *Col22a1*, *Gpr1*), 1 sublining fibroblast (*Thy1*, *Col1a1*, *Col1a2*), and 1 perivascular fibroblast (*Notch3*, *Myh11*, *Myl9*) (Figure 6, A–C). We named Prg4 lining fibroblasts because they line up along the surface of articular disc and synovial tissues in the TMJ (Figure 6D) and specifically expressed *Prg4*, encoding lubricin that is also known as Prg4 (Figure 6, B, C, and I). Thy1 sublining fibroblasts are named because they are located in the synovial sublining underneath Prg4 lining fibroblasts (Figure 6D) and have enriched expression of *Thy1* encoding CD90 (Figure 6I). UMAP analysis showed that Notch3 fibroblasts had a distinct location separated from lining and sublining fibroblasts and coexpressed *Acta2* (α-SMA) and *Myh11* (Figure 6I), which represent perivascular fibroblasts.

To examine synovial fibroblast dynamics during TMJOA, we performed RNAscope on TMJ sections at 3 weeks postinoculation of CFA or PBS. Prg4 lining fibroblasts had a distinct anatomic distribution at the surface of the articular disk and synovial tissues, whereas Prg4-immune reacting cells were significantly reduced at the anterior, posterior, and superior regions of joints (Figure 6, D–H). Prg4 produces lubricin and plays essential roles in boundary lubrication and joint function (45). Therefore, Prg4 lining fibroblast reduction is consistent with the cartilage remodeling and impaired TMJ function found in our CFA mice. Previous studies from knee RA suggest that Thy1 sublining fibroblasts undergo major expansion and drive the pathogenesis of joint inflammation and degeneration (44, 46). Similar to findings in knee RA, Thy1 fibroblasts were drastically increased at the anterior, posterior, and superior synovial tissue regions of the TMJ in CFA mice compared with controls (Figure 6, D–H). These expanded sublining fibroblasts can produce inflammatory cytokines, chemokines, growth factors, and neuropeptides, which likely contribute to the joint inflammation and peripheral sensitization in our painful TMJOA mice. Together (Figure 6J), our findings describe anatomically, molecularly, and functionally distinct fibroblast subsets that are dynamically changed in CFA inflammatory TMJOA with pain.

### Immune cell heterogeneity and anatomic position in TMJOA.

**Figure 7:**
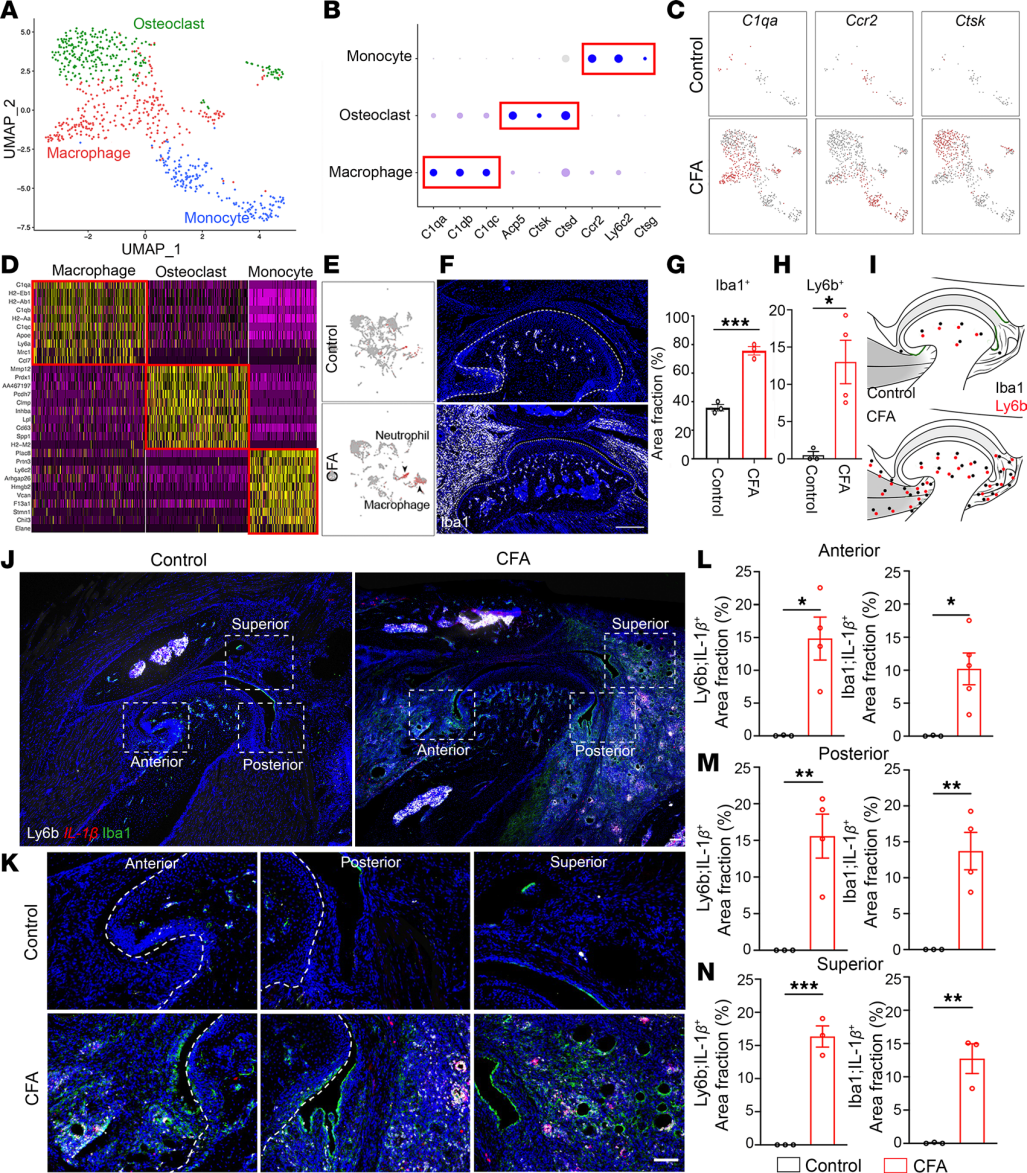
Immune cell heterogeneity and increased inflammatory response in CFA TMJ. (**A**) UMAP plot of immune cell clusters in adult mouse CFA TMJ. (**B** and **D**) Dot plot and heatmap of signature genes in different immune cell clusters including monocyte, osteoclast, and macrophage. (**C**) Feature plots showing the expression of indicated cell type marker genes that are overlaid on the UMAP plot. (**E**) UMAP plot for both control and CFA with the major cell types. Black arrowheads indicate the most upregulated cell types, including neutrophils and macrophages, in CFA TMJ. (**F**) Confocal imaging of sagittal TMJ sections stained with antibodies against Iba1 (white). DAPI stains nuclei (blue). Scale bar: 100 μm. (**G** and **H**) Quantification of the area fraction of Iba1^+^ macrophages (surrounding TMJ) and Ly6b^+^ neutrophils (anterior part of surrounding TMJ). *N* = 3–4 mice. (**I**) Schematic diagram of expanded Iba1^+^ macrophages (black) and Ly6b^+^ neutrophils (red) in CFA TMJ compared with control. (**J** and **K**) RNAscope of *IL-1β* (red) and immunofluorescence staining of Ly6b (white) and Iba1 (green) in different regions surrounding TMJ. DAPI stains nuclei (blue). Images in **K** (20× objective) are enlargements of boxed regions in **J** (4× objective). Scale bars: 100 μm. (**L**–**N**) Quantification of the area fraction of *IL-1β*^+^Ly6b^+^ and *IL-1β*^+^Iba1^+^ cells in the anterior, posterior, and superior regions of TMJ. *N* = 3–5 mice. All data are represented as mean ± SEM. Student’s *t* test. *N* = 3–5 mice, *P < 0.05, **P < 0.01, ***P < 0.001.

Inflammation is a well-accepted pathological factor for TMJOA with pain (44). To investigate the cellular basis of CFA-induced TMJ synovitis, we reclustered the immune cell populations and focused on neutrophils and 3 major macrophage-related cell subclusters, including macrophages, monocytes, and osteoclasts (Figure 7A). Analysis of enriched genes for each cluster allowed us to define macrophages (*C1qa*, *C1qb*, *C1qc*), macrophage precursor monocytes (*Ccr2*, *Ly6c2*, *Ctsg*), and macrophage-derived osteoclasts (*Acp5*, *Ctsk*, *Ctsd*) (Figure 7, A–D). We focused on neutrophils and macrophages because feature plots showed that these cells were mostly expanded in CFA mice compared with controls, including Ly6b neutrophils (Figure 7E) and 3 populations of macrophage-related cells (Figure 7C). We performed IHC staining of TMJs using Iba1 to label macrophages and Ly6b to label neutrophils. These studies verified a significant increase in the Iba1-positive macrophages and Ly6b-positive neutrophils in the anterior, posterior, and superior synovial tissues in CFA mice compared with controls (Figure 7, F–I).

To examine macrophage status, we used CD68 to monitor macrophage activation and found that these Iba1-positive macrophages were in an activation state. Importantly, there was a substantial increase in Iba1^+^CD68^+^ double-positive cells at the anterior and posterior synovium, as well as the RDT, of CFA mice compared with controls (Supplemental Figure 3, A–E), suggesting macrophage activation. The macrophages have been generally categorized into inflammatory M1 macrophages marked by the expression of *Il1β* and *Tnfα* or antiinflammatory M2 macrophages expressing *Arg1*, *Tgfb1*, and *Il4*. Interestingly, feature plots showed that all these increased macrophages, monocytes, and osteoclasts in CFA-inoculated TMJs expressed the combined inflammatory and anti-inflammatory cytokines, including *Il1β*, *Tnfα*, and *Tgfb1* (Supplemental Figure 3F), suggesting the heterogeneity of immune cells in the TMJ under OA conditions. To verify the inflammatory status of immune cells, we performed an RNAscope of *Il1β* inflammatory cytokines in conjunction with Iba1-labeled macrophages and Ly6b-labeled neutrophils. There was barely any *Il1β* detected in the control TMJ. In contrast, *Il1β* was drastically increased in Iba1-positive macrophages and Il6b-positive neutrophils in the CFA-inoculated TMJ at the anterior, posterior, and superior regions (Figure 7, J–N), which is consistent with synovitis and joint defects in CFA mice. Together, our findings reveal anatomically, molecularly, and functionally distinct immune cell populations with robust upregulation of inflammatory macrophages and neutrophils in TMJOA.

### The functional importance of Igf1 signaling in Cx3cr1 barrier macrophages in TMJOA.

**Figure 8:**
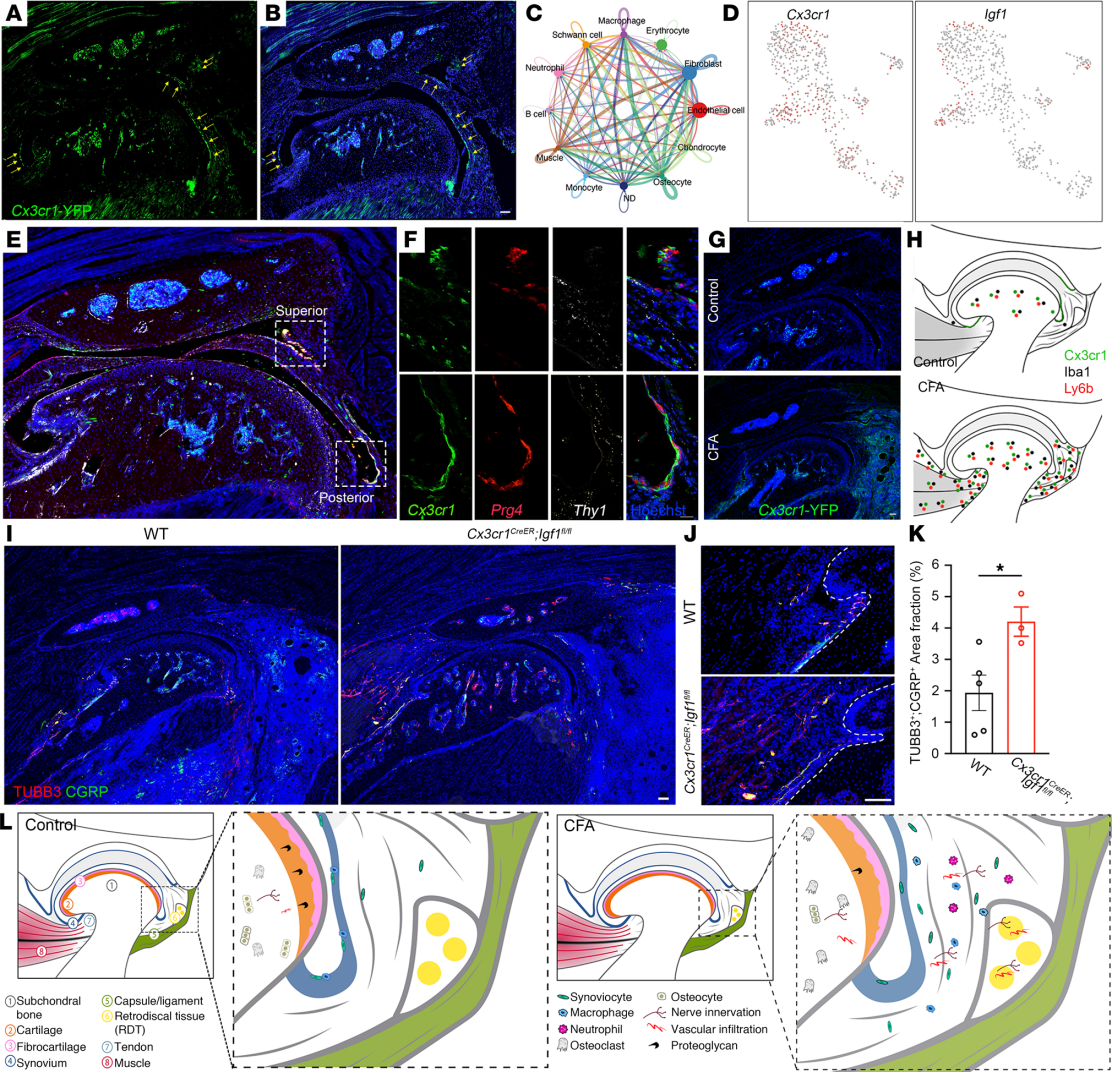
The functional importance of Igf1 signaling in barrier macrophages in TMJOA. (**A** and **B**) The expression of *Cx3cr1*-YFP at the synovial membranes. Arrows indicate Cx3cr1^+^ macrophage lining cells (green). DAPI stains nuclei. Scale bar: 100 μm. (**C**) NetVisual_circle plot for the cell-cell interaction between major cell types in the TMJ. (**D**) Feature plots showing the expression of *Cx3cr1* and *Igf1* genes that are overlaid on the UMAP plot of CFA TMJ. (**E** and **F**) Confocal imaging of sagittal TMJ sections stained with Cx3cr1-YFP (green) in conjunction with RNAscope of *Prg4* (red) and *Thy1* (white). DAPI stains nuclei (blue). Images in **F** (63× objective) are enlargements of boxed regions in image **E** (4× objective). Scale bars: 100 μm. (**G**) Confocal imaging of sagittal TMJ sections stained with Cx3cr1-YFP in control and CFA mice. DAPI stains nuclei (blue). Scale bar: 100 μm. (**H**) Schematic of Cx3cr1 cells together with Iba1 macrophages and Ly6b neutrophils in control and CFA TMJ. (**I** and **J**) Confocal imaging of sagittal whole TMJ or anterior TMJ sections stained with antibodies against TUBB3 (red) and CGRP (green). DAPI stains nuclei (blue). Scale bars: 100 μm. (**K**) Quantification of the TUBB3^+^CGRP^+^ area fraction. (**L**) A schematic diagram of dynamic changes in cell type and anatomical position in the TMJ under healthy conditions and TMJOA with pain. All data are represented as mean ± SEM. Student’s *t* test. *N* = 3–5 mice, *P < 0.05.

Previous studies suggest that a population of Cx3cr1-positive synovial macrophages provides a protective barrier for the knee joint (47). To investigate whether similar barrier macrophages occur in the TMJ and how they change in TMJOA, we used *Cx3cr1*–yellow fluorescent protein (*Cx3cr1*-YFP) knockin mice to genetically label synovial macrophages. A time-lapse movie of confocal imaging of the TMJ showed that *Cx3cr1*-YFP macrophages were enriched in the surface areas of the peripheral disc and the synovial membrane (Supplemental Video 4), which is consistent with barrier macrophages described in knee joints (47). Sectioned IHC staining verified the synovial lining and articular disc surface localization of C3xcr1-positive macrophages (Figure 8, A and B). We have shown that Prg4 lining fibroblasts and Thy1 sublining fibroblasts are also located in the synovial tissues (Figure 6). Next, we investigated the anatomical organization of barrier macrophages and fibroblasts. We performed RNAscope of *Prg4* and *Thy1* in the C3xcr1-YFP TMJ tissue sections and found that Cx3cr1-positive macrophages were located at the outermost layer toward the joint cavity, followed by the Prg4 lining fibroblasts, and then Thy1 sublining fibroblasts at the innermost layer within the synovial tissues (Figure 8, E and F), forming a sandwich-like synovial structure. Cx3cr1-positive cells were connected with each other to form a barrier-like morphology lining the synovial tissues, which were disrupted, leading to massively expanded Cx3cr1-positive macrophages in the CFA TMJ (Figure 8G). Feature plots showed that these expanded Cx3cr1-positive immune cells could be monocytes, macrophages, or osteoclasts (Figure 8D). Together, these results reveal disc and synovial barrier macrophages labeled by Cx3cr1, whose organization is disrupted, leading to Cx3cr1-positive immune cell expansion in TMJOA (Figure 8H).

The coexistence of expanded Thy1 fibroblasts and Cx3cr1 macrophages at the same synovial tissues of CFA TMJ prompted us to investigate cell-cell interaction. We analyzed our scRNA-Seq data with the CellChat program, which allows the prediction of ligand and receptor interactions at single-cell resolution (48). Numerous signaling interactions were found among different cell populations in the TMJ, reflecting their sophisticated interactions (Figure 8C). Igf1 signaling has been reported to regulate cartilage in TMJ as well as spinal cord neuropathic pain (49, 50), although its roles in cell-cell interaction of the TMJ and joint pain remain unknown. Therefore, we focused on Igf1 and found its enriched expression in Cx3cr1-positive macrophages (Figure 8D). To test functions of IGF signaling in cell-cell interaction in TMJOA, we generated *Cx3cr1^CreER^ Igf1^fl/fl^* mice in which the *Igf1* was deleted in macrophages. The results showed that *Igf1* depletion resulted in decreased CD206-positive M2 macrophages, suggesting that Igf1 is required for proper cell type and cell status in TMJOA. Importantly, IHC staining of sagittal TMJ sections showed that CFA mice exhibited an increase in neural activation marker CGRP-immune reacting area and induced innervation labeled by TUBB3 (Figure 8, I–K), which could be due to the increased inflammatory macrophages. Induced innervation in *Igf1* macrophage-specific deletion is consistent with pain behavior and suggests the functional importance of Igf1 signaling in cell-cell interaction among different cell types that are involved in TMJOA pain.

Our studies have identified molecularly distinct cell types, mapped their anatomical positions, defined cell morphological features, revealed signals mediating cell-cell interaction, and captured cell type and cell status dynamics in TMJOA with pain. The synovial membrane consists of a lining layer and sublining interstitial synovial tissue. The superficial lining surface is enriched with barrier macrophages and lining fibroblasts, whereas the sublining connective tissues contain nerves, blood vessels, lymphocytes, mast cells, adipocytes, and heterogeneous populations of interstitial macrophages and fibroblasts, all of which are subject to the dynamic changes leading to TMJOA (Figure 8L). This single-cell atlas provides a source to map the cell heterogeneity of the TMJ and to develop mechanism-based targeting for TMJOA with pain.

## Discussion

We have utilized an inflammatory TMJOA mouse model to recapitulate OA pathologies and pain in patients. Despite the well-recognized importance of animal models in TMDs, no one animal model is sufficient in mimicking the complex clinical conditions of TMJOA, as each model has its limitations and has different translatability to human clinical conditions (4, 5). It is challenging to combine several contributing factors into 1 animal model to replicate the multiple etiologies of TMJOA in patients. In addition to previously described pain behavior (20), our CFA-induced inflammatory mouse model exhibited OA-like phenotypes, subchondral bone loss, cartilage remodeling, and synovitis, which covers the major pathological features of TMJOA patients with pain. Moreover, the natural progression from localized TMJ arthritis to TMJOA can occur if there is no management to control contributing factors and stop disease progression. Therefore, our studies highlight inflammation as a driving factor of TMJ pain and are consistent with the notion that chemically induced inflammatory TMJ mice are suitable for OA and related orofacial pain (5, 24). Future studies should extend pain behavioral measurement into surgical models that are mainly used to investigate traumatic TMJOA.

Our findings suggest that CFA-induced TMJOA mice experienced severe pain at the early stage, followed by osseous and cartilage changes at the later stage. This observation is consistent with the notion that the disease initially starts with joint pain from capsulitis or synovitis and eventually ends with degenerative joint disease resulting in osteoarthritic changes (25, 51). Our cellular studies of TMJ degeneration cannot provide causative pain mechanisms, since pain is resolved at the stage when extensive joint degeneration occurs in our model. The temporal causative relationship between early pain and late joint remodeling remains to be established. As the disease progresses into the intermediate and late phases of TMJ degeneration, pain could subside, and bone remodeling could occur. It is possible that acute TMJ pain could result from CFA-induced localized inflammation, leading to peripheral sensitization. Without repeated CFA injection or persistent noxious stimuli to cause central sensitization, and with no impairment in pain modulation, there might be no pain behavior present at later stages. However, TMJ pain can be triggered again by abnormal joint loading and inflammation if the contributing factors related to excessive force and inflammation are not managed. Therefore, future studies in the pain process and the identification of potential therapeutic targets to reduce pain severity in the early phase, while preventing relapse and progression of the disease into the late phase of TMJ degeneration, would be beneficial for both TMJ arthritis and OA management.

Our studies revealed the anatomical pattern of joint innervation and neurovascular coupling in control and CFA TMJs. The joint synovium, capsule, RDT, subchondral bones, and muscle are innervated, which is consistent with reported nerve fibers immunoreactive for the CGRP (52, 53). It has been reported that knee joints are intensely innervated in subchondral bone (54, 55). The enriched innervation in synovial regions of the TMJ supports a synovium-driven peripheral sensitization model for orofacial pain in TMJs. In addition to encapsulated nerve endings, such as Ruffini-like receptors and the Pacini-like corpuscle (38), a free nerve ending plexus is found in the synovial regions and RDT, providing neural substrate for the orofacial pain in CFA mice. Future studies should identify genetic markers and functionally disturb specific sensory neuron subsets to confirm their roles in pain behavior. We showed that TMJ RDTs are highly innervated and well vascularized, containing intermingled Car4- and Plvap-positive capillary vessels. The RDT in the TMJ has been postulated to restrict pathological disc displacement, buffer mechanical force, and supply blood (56). We observed a robust colocalization of neurovascular bundles that are expanded in the anterior, posterior, and superior regions of the CFA-treated TMJ. The well-organized neurovascular structure is disorganized in CFA-treated TMJs. Future studies should investigate how pathological changes in RDT contribute to TMJ pain.

Our single-cell study provided a systematic view of cell diversity in the TMJ and their anatomical and dynamic changes in TMJOA. Recent single-cell studies focused on TMJ disc (57) or muscle (53, 58) under normal conditions, whereas our studies utilized the entire joint, with or without inflammatory OA, to provide a comprehensive analysis of all components in joint structures and to replicate the function of the TMJ as a whole. Furthermore, our volume image and multiplex immunodetection mapped the anatomical location of major cell types in TMJ under normal and pathological conditions. There is a robust expansion of immune cells in the CFA TMJ, including neutrophils, monocytes, macrophages, and osteoclasts. Increased osteoclasts might contribute to subchondral bone loss, whereas immune cell infiltration leads to synovitis and chondrocyte remodeling. These immune cells are heavily localized in synovial tissues as well as joint capsules and ligaments, regions innervated by nociceptive nerves. In addition, pro-inflammatory cytokine IL-1β–positive macrophages and neutrophils are drastically increased in CFA TMJs, suggesting an immune cell inflammatory status in addition to increased cell numbers. These observations highlight immune cells as pathological factors for TMJOA (5, 59). Similar to knee joints (47), we detected a layer of Cx3cr1-positive barrier macrophages in TMJs. Furthermore, we localized Cx3cr1-positive barrier macrophages to Prg4-labeled lining fibroblasts and Thy1-labeled sublining fibroblasts, which form a sandwich-like structure with potential cell-cell interactions. In CFA-induced inflammatory TMJs, the barrier-like structure is disrupted and coupled with drastic expansion of Cx3cr1-positive immune cells into the sublining region of synovium. It has been reported that Cx3cr1-positive cells could be bone marrow–derived osteoclast precursors with antiinflammatory functions (60), synovium-localized macrophage precursors with high osteoclast differentiation potential (61), and inflammatory osteoclasts with immune-suppressive functions (62). Future studies should determine the heterogeneity and function of these Cx3cr1-positive immune cells in the pathogenesis of TMJOA.

We found that fibroblast-like synoviocytes (FLSs) are robustly increased in CFA TMJs. These FLSs are composed of 3 subpopulations, including Prg4^+^ lining fibroblasts, Thy1^+^ sublining fibroblasts, and Notch3^+^ vessel-associated fibroblasts. Prg4 lubricin is substantially reduced in CFA TMJs at the anterior, posterior, and superior synovium. Considering OA-like defects in *Prg4*-knockout mice (45), these results suggest that *Prg4* deficiency and lining fibroblast disruption might contribute to OA-like defects in CFA mice. There is a drastic expansion of Thy1^+^ sublining fibroblasts in the CFA TMJ; Thy1^+^ sublining fibroblasts transform into a hyperplastic invasive tissue mass known as pannus. Therefore, TMJ synovitis and OA with pain could be due to a loss of normal functions of lining fibroblasts in lubricating cartilage and a toxic gain of function of sublining fibroblasts because of their interactions with immune cells and nociceptive nerves. Overall, FLS expansion and immune cell infiltration lead to synovial inflammation, angiogenesis, and peripheral nociceptive activation, which collectively contribute to pain. Our single-cell atlas should be used to investigate how specific cell types such as FLSs and macrophages within synovium might release signaling factors, chemicals, cytokines, and neurotrophic factors to activate nociceptors leading to pain hypersensitivity. Further functional studies of specific molecules and pathways as well as cell-cell interaction will provide potential therapeutic targets for TMJOA with pain.

## Methods

Further information can be found in Supplemental Methods.

### Sex as a biological variable.

Female mice were used in this study because of the presence of sexual dimorphism in the baseline nociceptive thresholds of rodents, with males and females potentially exhibiting distinct pain responses. In addition, female patients with TMD reported greater TMJ tenderness and a higher prevalence of the condition than male patients (63, 64).

### Mouse models.

The C57BL/6J (JAX#000664), *Thy1-*YFP (JAX#007788), *Cx3cr1^CreER^* (JAX#021160), *Igf1^fl/fl^* (JAX#012663), *Na_v_1.8-Cre* (JAX#036564), and *tdTomato* (JAX#007905) mouse strains were obtained from The Jackson Laboratory. Female 8- to 12-week-old mice were used in this study. The mice were housed in a controlled environment with specific pathogen–free conditions, including a 12-hour light/12-hour dark cycle, controlled temperature, and humidity. Euthanasia was conducted using carbon dioxide overdose, followed by cervical dislocation.

### CFA intra-articular injection.

The TMJ injection site is identified by palpating the zygomatic arch. At the posterior end of the zygomatic arch, there is a depressed area approximately 2 mm in front of the ear canal, which was used to guide the selection of the injection site. We inserted the needle into the injection site, which is under the posterior end of the zygomatic arch. The tip of the needle should contact the bone at a depth of ~2 mm.We slowly injected 10 μL of CFA (5 mg/mL; Chondrex, Inc) into the bilateral TMJ capsules and waited for at least 5 seconds before gradually withdrawing the needle. The control group received bilateral injections with 10 μL sterile PBS.

### Nociceptive behavior assessment.

The mice were acclimated to the testing environment for at least 1 hour before behavioral testing. The baseline measurements of bite force and head withdrawal threshold were recorded during the training period (>3 days) before starting to perform PBS/CFA intra-articular injection.

### Bite force measurement.

Each mouse was placed in a 50 mL plastic syringe, which had been modified to loosely restrain the mice while allowing the mouse’s head to move comfortably. Mice were trained for at least 3 days to bite and usually started to bite within 10 seconds after training. If the mice did not start biting within 10 seconds after the third training day, they were excluded from the experiment. Bite force is measured by a bite force transducer (YFM-1-100 Bite force sensor, 0-100N) connected to the NB IT RSD-V2.6.3 program (NST2000 data collector) from Nanjing Shen-yuan-sheng Intelligent Technology Co. The voluntary biting was recorded for 2 minutes per session, and the top 5 results of bite force amplitude were averaged for the quantification.

Head withdrawal threshold is measured by electronic von Frey analgesiometer. The mice were placed in a wire mesh cage and habituated in a behavioral room for at least 1 hour before the testing procedures. Von Frey filament was applied perpendicularly to the TMJ area. The head withdrawal threshold is defined as the minimum force from the von Frey filament that can elicit a withdrawal reflex. The measurement was recorded in at least 5 tests for each mouse at an interval of 10 seconds. The duration of the restraint period was approximately 2 minutes. The average of these values was identified as the head withdrawal threshold.

### Histology for OA and synovitis score.

The TMJs from both the CFA-injected and control groups were dissected; fixed in 10% formalin; decalcified in 14% EDTA (pH 7.2~7.4); processed in a series of ethanol solutions with concentrations of 70%, 80%, 95%, and 100%, followed by xylene; and finally embedded in paraffin. Histological sagittal sections of the TMJs in 5–8 μm were used for H&E staining to detect histological changes in joints. The severity of TMJOA was assessed using a modified OARSI scoring system (65). Scores ranged in each case from 0 to 6 points, where 0 indicated no evidence of osteoarthritis, and higher scores indicated a more severe osteoarthritis phenotype, characterized by unclear borders between cartilage and subchondral bone, uneven cartilage surfaces, and decreased hypertrophic layer thickness. Synovitis quantification followed a scoring system based on observations of synovial lining hyperplasia and inflammatory infiltrate (65). The grade of synovial lining hyperplasia ranged from 0 to 2. A score of 0 indicated a normal synovium structure (1–3 lining layers). A score of 1 was assigned to 4–6 lining layers, whereas a score of 2 was given for 7 or more layers. Inflammation was evaluated by an inflammation score, ranging from 0 to 3, based on the number and size of inflammatory infiltrates.

### Immunostaining.

Brain, TG, and TMJ sections were used for immunofluorescence staining following standard protocols. Brain and TG were isolated from the cranium and fixed with 4% paraformaldehyde at 4°C overnight. Then, samples were dehydrated with 30% sucrose at 4°C overnight, followed by 30% sucrose/OCT (Tissue-Tek, Sakura) (1:1) at 4°C overnight, and embedded with OCT on dry ice. For cryosections of the TMJ, the samples were decalcified in 14% EDTA for at least 10 days and dehydrated gradually in 15% sucrose for 2 hours, followed by 30% sucrose for 2 hours and 60% sucrose/OCT (1:1) at 4°C overnight. Then, samples were embedded in OCT compound, frozen on dry ice, and sectioned at 14 μm thickness using a cryostat (Leica CM1850).

Antigen unmasking solution (Vector Labs, H-3300) was used for antigen retrieval. The primary antibodies were as follows: Cathepsin K (Proteintech 11239-1-AP), CD31 (R&D Systems, Bio-Techne; AF3628), and βIII-Tubulin (Cell Signaling Technology; 5666S); Iba1 (FujiFilm, 019-19741), CGRP (Abcam; AB36001), CD68 (Bio-Rad; MCA1957GA), Ly6b (Bio-Rad; MCA771GT), CD206 (Abcam; ab64693), Car4 (R&D Systems, Bio-Techne; AF2414), Plvap (BD Pharmingen; 553849), and Alexa Fluor 488/568/647 (Invitrogen A-21206, A-11011, A-31573) were used as secondary antibodies. DAPI (Invitrogen; catalog 62248) was used for nuclear staining. The percentage of positive immunofluorescence signals and area fraction were determined using ImageJ software (NIH). Quantification was performed using 3 to 5 sections per mouse. Each dot in each graph represents the mean value of each sample or mouse within the group. At least 3 mice from independent mouse litters were analyzed for each group or genotype. Student’s 2-tailed *t* tests were used for statistical analysis. A significance level was set at a *P* value of 0.05.

### RNAscope staining.

Sample preparation and RNAscope staining were performed according to standard ACD (Bio-Techne) protocol with the RNAscope Multiplex Fluorescent v2 (catalog 323100). The probes used for this study were as follows: Mm-*Thy1* (430661-C1), Mm-*Prg4* (437661-C2), and Mm-*Il1b* (316891-C1). A negative control probe (probe 320871) was used for staining and imaging to minimize background signals. Quantification of RNAscope staining by ImageJ was performed at 20× original magnification and interpreted according to ACD scoring guidelines. At least 3 mice from independent litters were analyzed for each group or genotype. Student’s 2-tailed *t* tests were used for statistical analysis with a significance level *P* value of 0.05.

### scRNA-Seq and library preparation.

Female 8-week-old female mice were injected with CFA or PBS and were euthanized 3 weeks later. Perfusion was performed using 20 mL of cold PBS–diethylpyrocarbonate (PBS-DEPC). Tissues surrounding the TMJ structure were collected and dissected in PBS-DEPC on ice. TMJ tissues consisting of TMJ condyle, disc, temporal bone, muscle, and ligament surrounding the TMJ area were collected (*n* = 3) and minced into small pieces in tissue suspension medium containing MEM with 2% FBS. For each experimental group, TMJ tissues were transferred into a new 15 mL centrifuge tube containing 10 mL of tissue digestion medium (Collagenase P 1 mg/mL, Dispase II 2 mg/mL, MilliporeSigma). Tissues were digested while rotating at 37°C for 25 minutes. After digestion, tissues were resuspended with tissue suspension medium and centrifuged at 500*g* for 10 minutes at 4°C. Then, the supernatant was removed. Tissues were resuspended with 4 mL of DNase I solution (2 U/mL of DNase I in MEM) and incubated for 10 minutes at 37°C. Next, tissue suspension medium was added and resuspended on ice by pipetting several times to dissociate the tissue. After final dissociation by pipette, tissue samples were filtered through a 70 μm nylon mesh (VWR strainer) and centrifuged at 300*g* for 10 minutes at 4°C.

Immune cell depletion was performed as described in MojoSort Mouse CD45 Nanobeads Protocol – Depletion (BioLegend). Briefly, samples from the cell dissociation step were washed and resuspended with cold MojoSort Buffer (BioLegend, catalog 480017). Cells were pelleted via centrifugation at 300*g* for 5 minutes at 4°C, and the supernatant was discarded. Cell viability counts were performed before resuspending the cells in an appropriate volume of buffer to adjust the concentration to 1 × 10^8^ cells/mL. Then, 100 μL of the cell suspensions was aliquoted into a new tube. A total of 10 μL of MojoSort Mouse CD45 Nanobeads (BioLegend, catalog 480027) was added for 1 × 10^7^ cells/mL and incubated on ice for 15 minutes. Samples were washed with MojoSort Buffer and centrifuged at 300*g* for 5 minutes. The supernatant was then discarded, and the cells were resuspended in 2.5 mL of MojoSort Buffer. The tube was placed in the MojoSort Magnet (BioLegend, catalog 480019) for 5 minutes. The CD45^+^ cell depletion was repeated by pouring out the unlabeled fraction into the new tubes and placing the tube in the magnet for another 5 minutes. After immune depletion, final cell counts were performed to prepare the cell suspension for scRNA-Seq using a Chromium Controller (10x Genomics). Approximately 16,500 cells were loaded into the Chromium system with a targeted recovery of 10,000 cells. Library preparation was performed following the manufacturer’s protocol, for Chromium Next GEM Single Cell 3’ Reagent Kits v3.1 (Dual Index) (10x Genomics), and sequencing was conducted using NovaSeq X Plus (Illumina).

### scRNA-Seq analysis.

Quality control of raw reads was performed with 10x Genomics Cell Ranger 7.0.1. Cell Ranger Count (10x Genomics) was used to align samples to the reference genome GRCm38 (mm10), quantify reads, and filter reads and barcodes. Data analysis was performed with Seurat version 4.9.9. Cells were filtered to exclude cells with fewer than 500 genes or more than 10% mitochondrial content. Filtered cells were normalized with the SCTransform method. Datasets of PBS and CFA were integrated using the FindIntegrationAnchors function. Principal component analysis was performed, and the top 200 principal components were selected for dimensionality reduction using the UMAP algorithm. Clustering was performed with the FindCluster function with a resolution of 1.5. Marker genes were identified by comparing each cluster against all other clusters using the FindAllMarkers function with optimized setting (log fold-change threshold of 0.25 and >25% cells expressing the gene).

### Statistics.

Statistical analyses were performed using GraphPad Prism (version 9.0.0) software. Data are presented as mean values ± SEM. To compare more than 2 experimental groups, a 1-way ANOVA with Tukey’s post hoc test was performed, and for comparison between 2 groups, 2-tailed unpaired Student’s *t* test was used to calculate *P* values. *P* < 0.05 was considered statistically significant. Each experiment was repeated independently at least 3 times with similar results as specified in figures and figure legends. Graphs showing mean ± SEM for each group represent at least 3 biological replicates (*n* ≥ 3 mice, as indicated in figure legends). Images of immunofluorescence staining were representative of at least 3 biological replicates (*n* ≥ 3 mice) showing similar results.

### Study approval.

All studies were performed with the approval of the Institutional Animal Care and Use Committee of the University of Southern California, Los Angeles, California, USA.

### Data availability.

The scRNA-Seq data generated in this study have been deposited in National Center for Biotechnology Information Gene Expression Omnibus under accession number GSE267942. Supporting Data Values are provided with this paper.

## Author contributions

SJ, YS, ZL, JC, QC, and PFK performed all experiments. ZL analyzed all bioinformatic data. JFC designed the experiments and supervised the research. JFC wrote the manuscript with assistance from SJ and YS.

## Supplementary Material

Supplemental data

Supplemental video 1

Supplemental video 2

Supplemental video 3

Supplemental video 4

Supporting data values

## Version 1 02/10/2025

Electronic publication
