# Supplementary material for: Mapping cell diversity and dynamics in inflammatory temporomandibular joint osteoarthritis with pain at single-cell resolution
Source: JCI Insight. 2025 Feb 10;10(3):e184379. doi: 10.1172/jci.insight.184379 (PMC11948589; doi:10.1172/jci.insight.184379)
Supplement: Supplemental data [file jciinsight-10-184379-s048.pdf]

Supplemental Materials and Methods

Micro-CT (µCT) analysis

Micro-CT live imaging was performed on a Scanco Medical μCT 50 scanner (Scanco Medical, Switzerland) at the University of Southern California Molecular Imaging Center (90 kVp, 78 µA, 10 µm pixel size). AVIZO 9.4.0 (Thermo Fisher Scientific) was used to perform 3D reconstruction of the TMJ. The microarchitecture parameters of the subchondral bone, including bone volume over total volume (BV/TV), trabecular spacing (TbSp), trabecular thickness (TbTh), and trabecular number (TbN) were analyzed using VGStudio Max3.3 (Volume Graphics, Inc., USA). For each sample, three spherical regions of interest (ROI) with a radius of 0.1 mm were selected at the midpoints of the anterior, middle, and posterior condyle for all measurements. Then, the values of each bone parameter listed above were averaged. At least 4 mice from independent mouse litters were used to calculate each parameter. Each dot in the graph quantification represents one sample (n). Student's *t*-test was used for statistical analysis. A significant level was set at *p*-value of 0.05.

CUBIC clearing

The animals were thoroughly anesthetized and then subjected to transcardiac perfusion using PBS followed by 4% PFA. After perfusion, the TMJs were carefully dissected and preserved by 4% PFA for 24 hours at 4°C. The TMJs were rinsed with PBS and then decalcified in 14% EDTA (pH 7.4) for 7 days at 4°C. Next, the decalcified TMJs were dehydrated using 30% sucrose. After dehydration, the TMJs were rapidly frozen using Tissue-Plus O.C.T. compound. The embedded TMJs were sectioned using a cryostat (Leica, Cat #CM1950) at a thickness of 100 µm. The slides were performed using CUBIC clearing as in the following method. The tissues were treated with Tissue-Clearing Reagent CUBIC-L (TCI, T3740) and incubated at 37°C for 24 hours. After three washes with PBS, the samples were then placed in Tissue-Clearing Reagent CUBIC-R (TCI, T3741) and left overnight at 37°C until they were transparent. The slides were prepared for imaging by mounting them with CUBIC-R solution.

iDISCO tissue clearing

The adult mice were euthanized using carbon dioxide (CO2) and then perfused with PBS, followed by 4% PFA/PBS for fixation. The TMJ samples were post-fixed overnight at 4°C. The fixed samples underwent two washes in PBS for 1 hour each. Subsequently, they were dehydrated in 30% ethanol (in ddH_2O) for 1 hour, followed by 1 hour in 50% ethanol, 80% ethanol, and 100% ethanol, twice each. The samples were then treated with 5% H_2O_2 (Sigma, H1009) (1 part 30% H_2O_2 mixed with 5 parts ice-cold ethanol) at 4°C overnight. After bleaching, the samples were gradually rehydrated in a series of ethanol and water solutions: 80% ethanol in H_2O for 1 hour, 50% ethanol in H_2O for 1 hour, 30% ethanol in H_2O for 1 hour, and finally in PBS for 1 hour, repeated twice. The samples were treated with a cold permeabilization solution consisting of 25% (w/w) Urea, 15% (w/w) Glycerol, 15% (w/w) Triton X-100, and 45% (w/v) double distilled water at 4°C for 5 hours. The samples were treated with enzyme-based digestion using 0.2% (w/v) Collagenase (Merck, 10,103,578,001) in PBS at 37°C for 30 minutes with continuous shaking. The samples were then rinsed twice for 5 minutes each using a wash solution consisting of 2% (v/v) FBS (Sigma-Aldrich, F7524) in PBS on a rocking shaker. The samples were transferred to a fresh blocking solution consisting of 10% (v/v) donkey serum (Abcam, ab7475), 10% (v/v) DMSO (Sigma-Aldrich, D5879), and 0.5% (v/v) Triton X-100 in PBS at 37°C for 20 minutes. After blocking, the tissues were further treated with the anti-RFP antibody (Rockland, 48776) in the

antibody dilution buffer containing 2% (v/v) donkey serum, 10% (v/v) DMSO, and 0.5% (v/v) Triton X-100 in PBS. The tissues were incubated overnight at 37°C in a rocking shaker at 120 rpm. After incubation, the samples were washed with washing solution containing 2% (volume/volume) donkey serum and 0.5% (volume/volume) Triton X-100 in PBS for 3 hours at 37°C in a rocking shaker at 120 rpm. The solution changed every 15 minutes for the first hour and then at every 30 minutes until finished. The secondary antibody was mixed with antibody dilution buffer. After incubation, the samples were washed according to the same washing process for the primary antibody. Following immunostaining, the tissues were dehydrated using a gradient of ethanol concentrations (30%, 50%, and 80%) for 30 minutes each, with gentle rotation at room temperature. The samples were then transferred to pure methanol for 1 hour, with the methanol being replaced with ethanol every 20 minutes. The methanol was then eliminated, and the samples were washed twice with Ethyl cinnamate (ECi) (Sigma-Aldrich, 112,372) for 5 minutes each. The bones were then treated with a clearing solution consisting of 80% (v/v) ECi and 20% (v/v) polyethylene glycol (PEG) (Sigma, 447,943) while gently rotating at room temperature for 30-60 minutes.

Confocal imaging and image processing

The cleared samples were imaged using a confocal microscope (Leica STELLARIS 5 Confocal Microscope Platforms) in a sagittal orientation, with the right lateral side facing downwards. The imaging was performed using a 10x/NA0.3 objective lens with a working distance of 2 mm, as well as a 63x/NA1.40 objective lens with a working distance of 200 µm. The LAS X Life Science Microscope Software (version 1.4.6) was utilized for acquiring images. The microscope is equipped with fixed laser lights at wavelengths of 405 nm, 488 nm, 561 nm, and 633 nm. The scans were conducted at a zoom factor of 0.75x using either a 10x/NA0.3 or 63x/NA1.40 objective lens. The samples were scanned with a step size of 1 µm, utilizing continuous scanning under 488 nm and 561 nm channels. To enhance the visual representation of the figures, a gamma correction was applied to the raw data acquired from the Confocal Microscope. The process of converting file formats was carried out using ImageJ (NIH, http://imagej.nih.gov/ij). Bitplane Imaris (http://www.bitplane.com/imaris/imaris, version 10.1.1) was used for doing 3D reconstructions, manual 3D annotations, and generating movies.

Supplemental Figure Legends

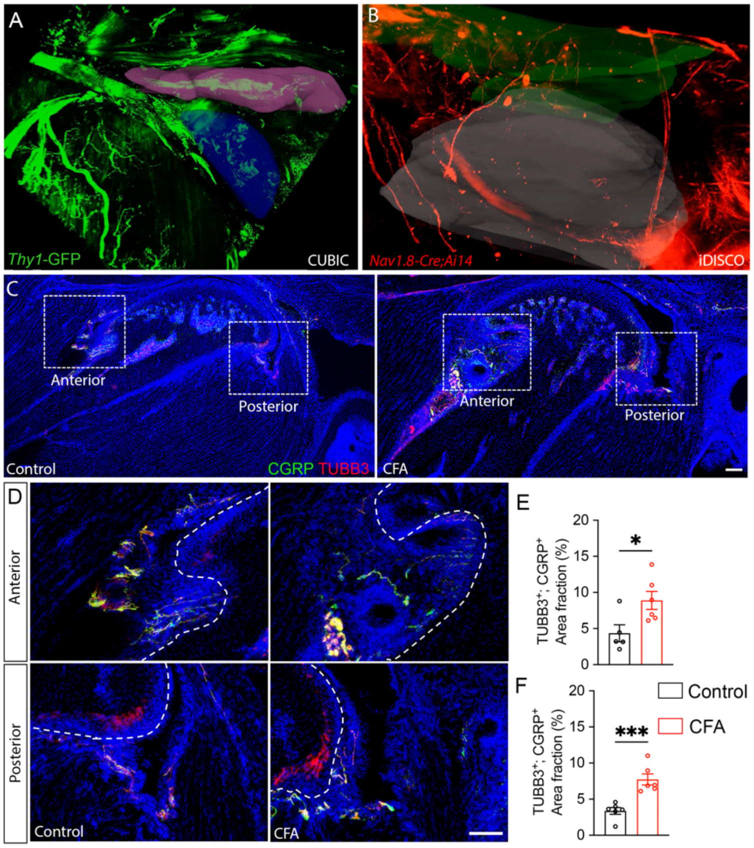

Figure S1. Characterization of TMJ innervation in control and CFA mice. (A) Confocal imaging of TMJ from adult *Thy1*-GFP mice after CUBIC tissue clearance. (B) Confocal image of TMJ from adult *$Na_V1.8$-Cre;Ai14* mice after iDISCO tissue clearance. (C, D) Immunofluorescence staining of anterior and posterior regions of sagittal TMJ sections with antibodies against CGRP (green) and TUBB3 (red). DAPI stains nuclei (blue). Scale bar: 100 µm. Images in D (20 x objective) are enlargements of boxed regions in C (4 x objective). (E, F) Quantification of $CGRP^+$;$TUBB3^+$ area fraction surrounding TMJ. N = 5-6 mice.

All data are represented as mean ± SEM calculated by Student's *t*-test, n = 5-6 mice, *$p<0.05$, ***$p<0.001$.

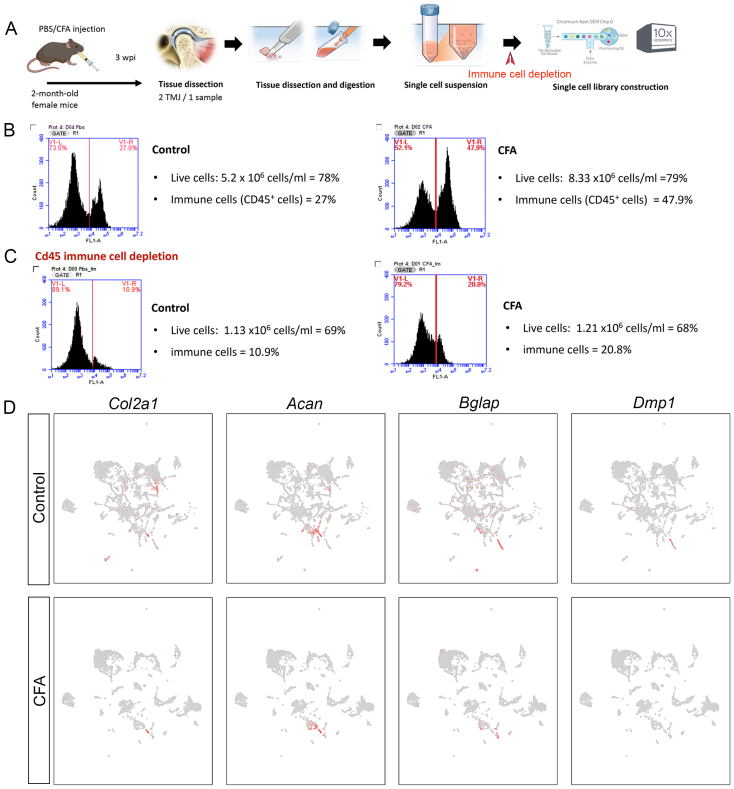

Figure S2. scRNA-seq analysis of TMJ in control and CFA mice. (A) Schematic diagram of TMJ inoculation and experimental procedure of single-cell analysis with immune cell depletion. (B, C) FACS analysis of live cells and immune cells before and after the Cd45 immune cell depletion in single-cell preparation. (D). Feature plots showing the expression of indicated genes that are overlaid on the UMAP plot.

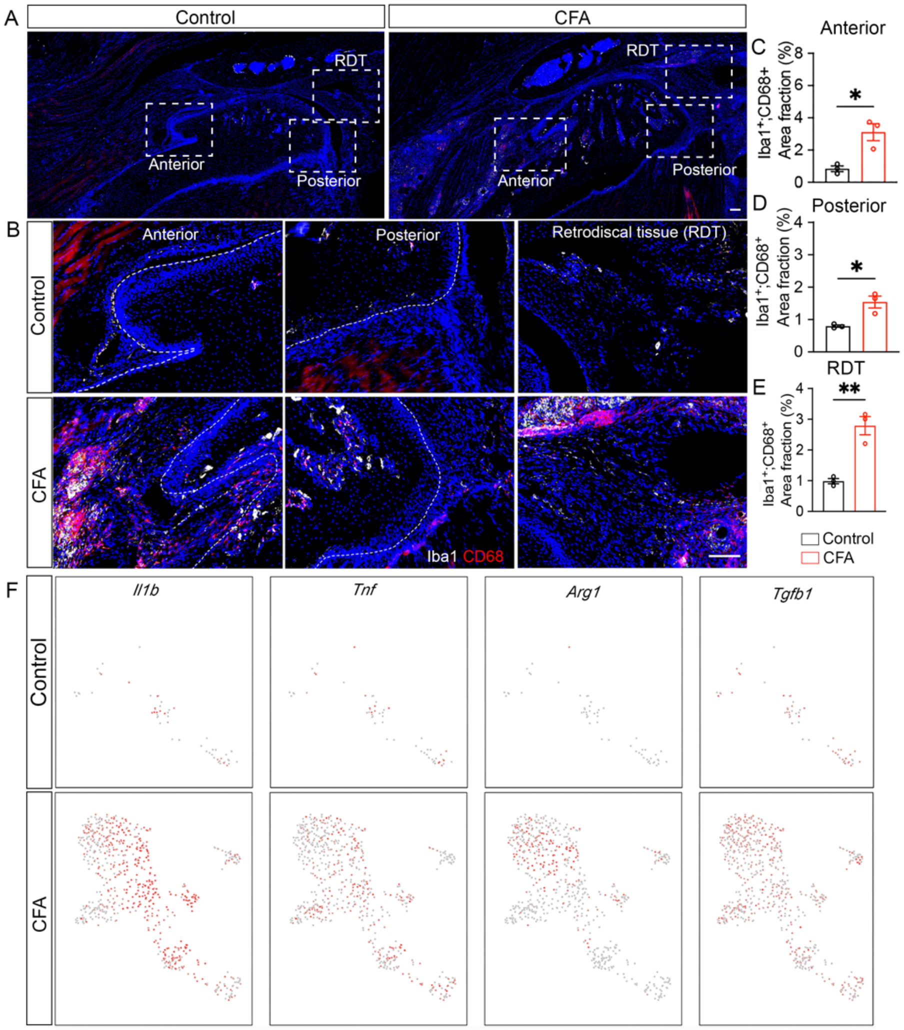

Figure S3. Macrophage activation in CFA TMJ. (A, B) Confocal imaging of sagittal TMJ sections stained with antibodies against Iba1 (red) and CD68 (white). DAPI stains nuclei (blue). Images in b (20 x objective) are enlargements of boxed regions of TMJ in a (4 x objective) at the anterior, posterior, and RDT regions. Scale bars: 100 µm. (C-E) Quantification of the area fraction of Iba1^{+};CD68^{+} in the TMJ area. (F) Feature plots showing the expression of indicated genes that are overlaid on the UMAP plot.

All data are represented as mean ± SEM calculated by Student's *t*-test, n = 3 mice, *p<0.05, **p<0.01.
